# Automated detection, segmentation and measurement of major vessels and the trachea in CT pulmonary angiography

**DOI:** 10.1038/s41598-023-45509-1

**Published:** 2023-10-27

**Authors:** Ali T. Kahraman, Tomas Fröding, Dimitrios Toumpanakis, Nataša Sladoje, Tobias Sjöblom

**Affiliations:** 1 Department of Immunology, Genetics and Pathology, Uppsala University, 751 85 Uppsala, Sweden; 2 Department of Radiology, Nyköping Hospital, 611 39 Nyköping, Sweden; 3 Department of Radiology, Uppsala University Hospital, 751 85 Uppsala, Sweden; 4 Department of Surgical Sciences, Uppsala University, 751 85 Uppsala, Sweden; 5 Centre for Image Analysis, Department of Information Technology, Uppsala University, 751 05 Uppsala, Sweden

**Keywords:** Cardiovascular diseases, Medical research

## Abstract

Mediastinal structure measurements are important for the radiologist’s review of computed tomography pulmonary angiography (CTPA) examinations. In the reporting process, radiologists make measurements of diameters, volumes, and organ densities for image quality assessment and risk stratification. However, manual measurement of these features is time consuming. Here, we sought to develop a time-saving automated algorithm that can accurately detect, segment and measure mediastinal structures in routine clinical CTPA examinations. In this study, 700 CTPA examinations collected and annotated. Of these, a training set of 180 examinations were used to develop a fully automated deterministic algorithm. On the test set of 520 examinations, two radiologists validated the detection and segmentation performance quantitatively, and ground truth was annotated to validate the measurement performance. External validation was performed in 47 CTPAs from two independent datasets. The system had 86–100% detection and segmentation accuracy in the different tasks. The automatic measurements correlated well to those of the radiologist (Pearson’s r 0.68–0.99). Taken together, the fully automated algorithm accurately detected, segmented, and measured mediastinal structures in routine CTPA examinations having an adequate representation of common artifacts and medical conditions.

## Introduction

Computed tomography (CT) pulmonary angiography (CTPA) is a medical imaging procedure where an intravenous (IV) contrast agent is used to enhance visualization of the thoracic blood vessels, in particular the pulmonary arteries^1^. The CTPA is the current reference imaging method for diagnosis of pulmonary embolism (PE)^2,3^, a condition associated with high mortality and morbidity that in the US alone affects more than 600,000 patients per year and causes > 100,000 deaths annually^4,5^. In the PE CTPA reading and reporting process radiologists manually perform quality control and different measurements of mediastinal structures in the imagery depending on the clinical situation and local tradition. Measurements of standard deviation (SD) of Hounsfield Units (HU) in a given region of interest (ROI) such as the descending aorta (DAo) and mean value of HU in the pulmonary trunk (PT), can be used for image quality assessment^6,7^. The mean value of HU in PT can help choosing an optimal window setting for PE detection^8^. Ascending aorta (AAo) and PT diameters could alert the radiologist to an aneurysm^9,10^ or possible pulmonary hypertension^11–14^. Performing these manual measurements is time-consuming^15^, and fully automatic solutions could therefore be of value to the radiologist.

Automated measurements of mediastinal structures demand accurate algorithms for detection and segmentation. Several solutions have been proposed to semi-automatically segment and measure mediastinal structures in CTPA examinations^16–18^. Deterministic approaches such as model-based^18^ and iterative^19^ methods for segmenting large vessels in CTPA examinations have been demonstrated. More recently, probabilistic approaches such as deep learning based systems have emerged^17,20^. However, these studies suffer from two main limitations in their datasets; they either (i) contain only a small number of examinations or (ii) lack examinations having the artifacts and co-morbidities encountered in daily radiology practice. Deep learning-based approaches have been trained and tested with a large number of CTPA examinations, but there is insufficient information on dataset characteristics^21^. To overcome these limitations, there is a need to train and test algorithms on large datasets with examinations of different image quality containing representative artifacts and medical conditions often encountered in radiology practice. Hence, in this study, we aimed to develop an algorithm that can accurately detect, segment and measure mediastinal structures in routine clinical CTPA examinations and benchmark its performance to the radiologist.

## Results

### Characteristics of study sample

A total of 700 CTPA examinations performed on 652 patients (54% women) referred because of clinically suspected PE between 2014 and 2018 were included. The age range was 16–100 years (median 72; interquartile range 18) and 25 female and 16 male patients underwent examinations twice, 1 female and 1 male patient underwent three examinations, and 1 female patient underwent four examinations (Table 1). The examinations were performed using five different CT scanners from three different manufacturers (Table 2). Ground truth for the measurements were generated through a comprehensive reading by the senior radiologist (TF) and the radiology resident (DT). For system development, 180 CTPA exams were randomly assigned to a training set and 520 were used for testing. Following quality scoring by the radiologist, 65% of training and 71% of test set cases, were of good or acceptable quality, whereas 35% and 29%, respectively, were classified as of inferior quality. No examinations were excluded.

**Table 1:** Radiological characteristics of 700 CTPA examinations used in CADe system training and testing.

Characteristic	Training Dataset	Test Dataset	All
Source	NH^a^	NH^a^	NH^a^
Study period	2014 to 2018		
No. of patients	170	482	652
No. of CTPA exams	180	520	700
Slice thickness (mm)			
0.625	114	193	307
0.9	10	150	160
1	39	167	206
2	17	10	27
Median slice numbers and IQR	468 (123)	479 (181)	476 (160)
Gender (women/men)	86/84	267/215	353/299
Median age and IQR (years)	72 (19)	72 (18)	72 (18)
Age range	17–93	16–100	16–100
Quality of CTPA exams			
Motion-breathing artifacts (mild/moderate/severe)	82/57/41	289/111/120	371/168/161
Streak artifacts (mild/moderate/severe)^b^	71/68/41	213/210/97	284/278/138
Median image noise (in 2.0 mm) and IQR (HU)^c^	23 (7)	19 (9)	20 (9)
Median image noise (in 0.625, 0.9 or 1.0 mm) and IQR (HU)^c^	28 (9)	27 (9)	27 (10)
Lung diseases, which affect quality^d^	82	306	388
Low contrast concentration (< 200 HU) in PT^e^	9	20	29
Calculated Quality score (good/acceptable/inferior)^f^	58/59/63	200/169/151	258/228/214
Manual measurement^h^			
Median IV contrast concentration in PT and IQR (HU)^e^	386 (166)	385 (156)	385 (158)
Median diameter of AAo and IQR (mm)	33 (6)	34 (6)	33 (6)
Median diameter of PT and IQR (mm)	27 (5)	27 (6)	27 (6)
Median ventricle ratio and IQR (mm)	0.93 (0.23)	0.95 (0.28)	0.95 (0.28)
Medical conditions			
Emboli present (in CTPA)	43	106	149
Emboli in (right/left) lung	38/35	99/79	137/114
Pneumothorax in (right/left) lung	0/0	4/2	4/2
Pleural effusion in (right/left) lung	66/62	164/152	230/214
Infiltrate or atelectasis (or other opacities) in (right/left) lung	110/92	334/321	444/413
Pericardial fluid (more than normal amounts)	23	60	83
Sign indicative of congestive heart failure^i^	33	98	131
Lymph nodes (mediastinum/right hilum/left hilum)^j^	24/17/12	114/59/42	138/76/54

Unless otherwise indicated, data are number of CTPA examinations and data in parentheses are interquartile range.

*CTPA* computed tomography pulmonary angiography, *HU* Hounsfield unit, *IV* intravenous contrast, *IQR* interquartile range, *PT* pulmonary trunk, ROI region of interest.

^a^Nyköping Hospital (NH), Sweden. ^b^Streak artifacts from superior vena cava affecting visualization of pulmonary arteries, AAo and other major structures of mediastinum. ^c^Measurement was done by calculating SD of HU in circular ROI of 1 cm^2^ in descending aorta at level of pulmonary trunk. ^d^Lung parenchyma pathology adjacent to pulmonary arteries complicating the evaluation of pulmonary embolism for the radiologists. ^e^Measurement was done by circular ROI of 2 cm^2^ in the PT, in axial plane with 2.0 mm slice thickness. ^f^Quality score calculation is described in Supplemental materials. ^h^Measurement of diameter of PT and ascending aorta was done at the level of the PT, in axial plane. ^i^Typical interstitial edema or typical ground glass (dependent parts of lobes). ^j^Lymph nodes with short axis > 1 cm in axial plane.

**Table 2:** Acquisition parameters for CT scanners.

Parameter	Brilliance 64	Ingenuity core	Ingenuity CT	LightSpeed VCT	Somatom definition flash
Manufacturer	Philips	Philips	Philips	General electric	Siemens
No. of CTPA exams^a^	81 (11.5)	142 (20.3)	18 (2.6)	326 (46.6)	133 (19)
Voltage (kVp)^a^					
80	4 (0.5)	5 (0.7)	–	21 (3)	122 (17.4)
100	77 (11)	131 (18.7)	18 (2.6)	299 (42.7)	5 (0.7)
120	–	6 (0.9)	–	5 (0.7)	6 (0.9)
140	–	–	–	1 (0.2)	–
Exposure time (ms)	711 (0)	627 (275)	368 (11)	500 (0)	285 (0)
Contrast media (ml)	40–100	40–100	40–100	40–100	40–100
Slice thickness (mm)					
0.625	–	–	–	307 (43.9)	–
0.9	–	142 (20.3)	18 (2.6)	–	–
1	73 (10.4)	–	–	–	133 (19)
2	8 (1.1)	–	–	19 (2.7)	–
Axial pixel size (mm)^b^	0.7 (0.04)	0.68 (0.06)	0.68 (0.02)	0.7 (0.04)	0.74 (0.1)
Reconstruction matrices^c^	512 × 512	512 × 512	512 × 512	512 × 512	512 × 512

Unless otherwise indicated, data are number of examinations. *CTPA* computed tomography pulmonary angiography, *kVp* peak kilo voltage.

^a^Data in parentheses are percentages.

^b^Median and interquartile range.

^c^Data are number of pixels.

### Detection and segmentation performance of the CADe system

**Figure 1:**
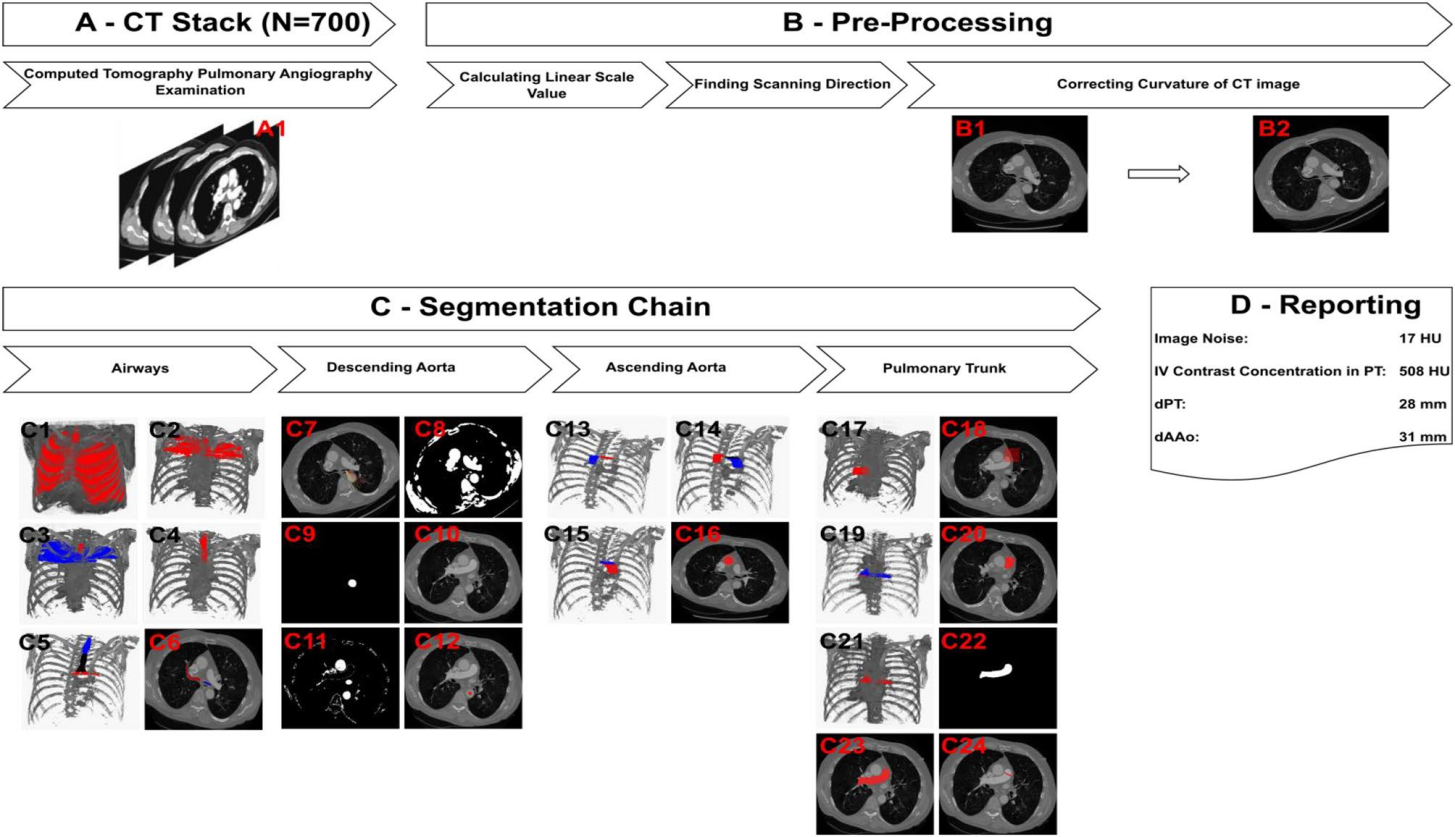
Flowchart of the CADe algorithm. (**A**) A total of 700 2 mm axial CTPA image stacks were exported from the PACS server. (**B**) In the pre-processing, the linear scale value and the curvature of the CT image were calculated. (**C**) The segmentation chain consisted of four steps, starting with trachea detection followed by DAo, AAo, and PT detection. (**D**) Noise assessment and measurements of mediastinal vascular structures were reported by the system. Graphical output of the system is shown in Supplemental Fig. 15.

**Figure 2:**
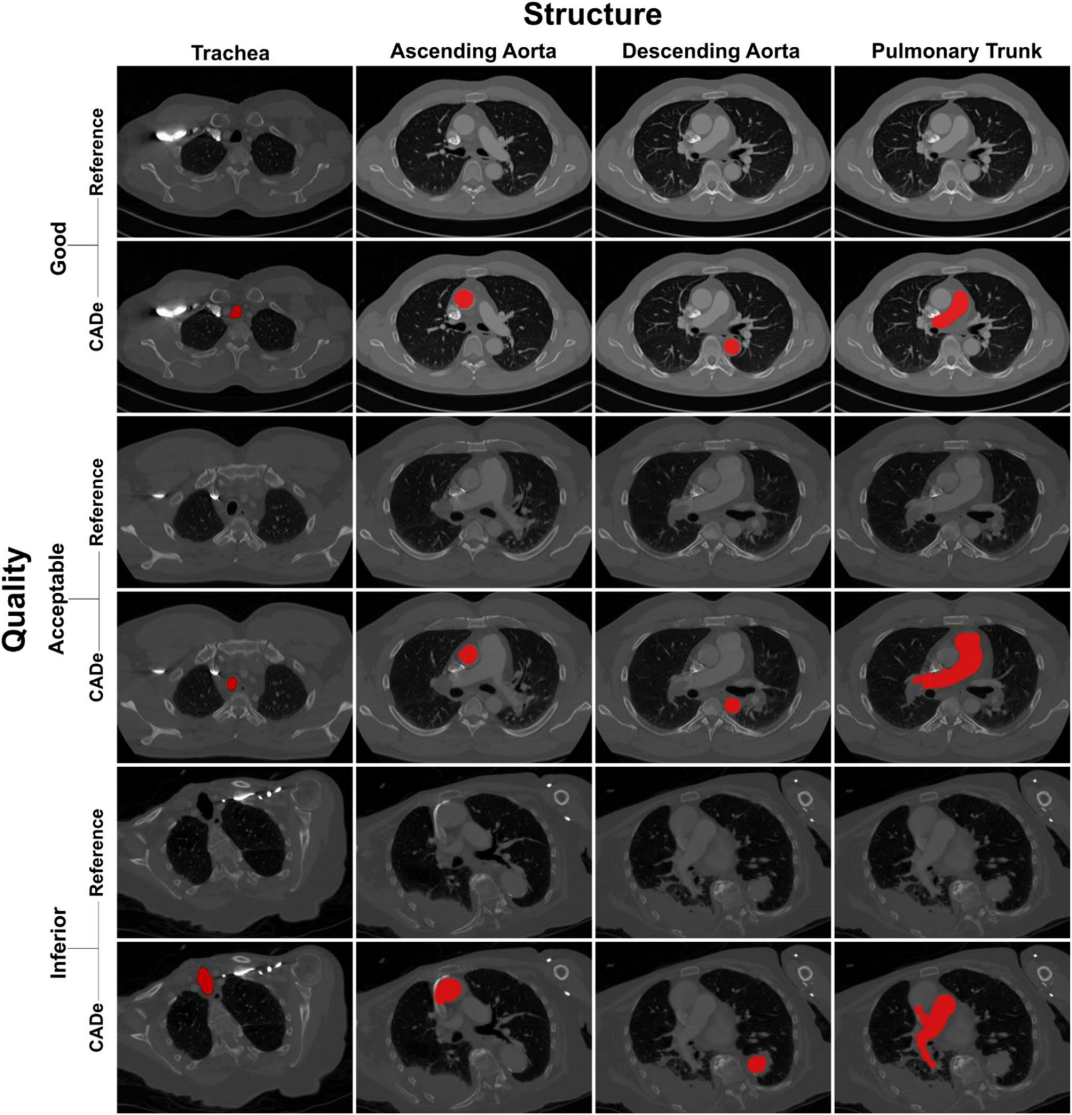
Automatic segmentation of trachea and vascular structures in CTPA examinations. Segmentation results of the CADe software (red) in representative CTPA examinations deemed by the radiologist as of good, acceptable or inferior quality are shown in red.

**Figure 3:**
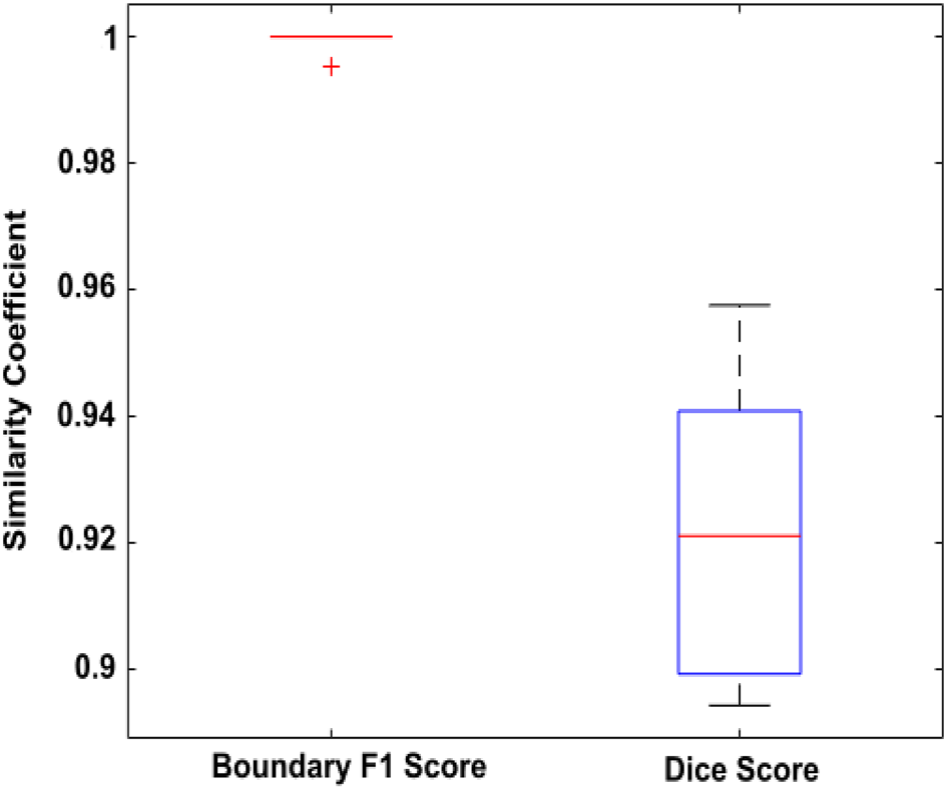
External validation of automatic AAo segmentation measurement. Boundary F1 and dice scores of the proposed CADe system for AAo segmentation in the SegTHOR dataset (n = 12 CTPA exams). Median (red line), interquartile range (boxes), and outliers ( +).

A fully automatic system for detection, segmentation and measurement of mediastinal structures was developed (Figs. 1 and Supplemental Fig. 1). It was then trained using 180 CTPA examinations and tested on 520 CTPA examinations. The detection and segmentation outputs from both the training and the test datasets were independently evaluated by two radiologists (Table 3) with high initial inter-observer agreement (99.52–100%). For the very few cases where there was a disagreement, a reevaluation was made by both radiologists to reach a consensus evaluation. Evaluation of anatomical landmark detection in test cases showed that the tracheal bifurcation and the carina of the trachea were correctly located in 96% of the examinations while the pulmonary vein (PV)/proximal part of the PT was correctly located in 87%. Similarly, for cardiovascular structures, the DAo was detected in 90%, the AAo was detected in 86%, and the PT was detected in 88% of the test set examinations. Once compartments were correctly detected (Supplemental Fig. 16), a quantitative evaluation revealed successful segmentation of the trachea, the DAo, the AAo and the PT in 100% of the test cases. When analyzed by examination quality, the tracheal bifurcation and the carina of the trachea were correctly detected in 97%, 97%, and 93%, and the PV/proximal part of the PT was correctly detected in 90%, 86%, and 85% of good, acceptable and inferior quality CTPA exams, respectively. The DAo was correctly detected in 92%, 91%, and 89%, the AAo in 88%, 85%, and 85%, and the PT in 90%, 86%, and 86% of good, acceptable and inferior quality examinations, respectively (Fig. 2). External validation of the AAo segmentation was performed in 12 CTPAs from the SegTHOR dataset^22^, where the median Dice score between the CADe system and manual segmentation was 0.92 ± 0.02 SD and the median Boundary F1 (BF) contour matching score was 1.0 (Fig. 3).

**Table 3:** Detection and segmentation task performance of CADe algorithm on the training and test data sets compared to ground truth.

Compartment	Training set				Test set				All (n = 700)
	Quality of CTPA exams				Quality of CTPA exams				
	Good (n = 58)	Acceptable (n = 59)	Inferior (n = 63)	All (n = 180)	Good (n = 200)	Acceptable (n = 169)	Inferior (n = 151)	All (n = 520)	
Trachea									
Detection	58 (100%)	59 (100%)	62 (98%)	179 (99%)	198 (99%)	168 (99%)	147 (97%)	513 (99%)	692 (99%)
Segmentation	58 (100%)	59 (100%)	62 (98%)	179 (99%)	198 (99%)	168 (99%)	147 (97%)	513 (99%)	692 (99%)
Tracheal bifurcation									
Detection	58 (100%)	59 (100%)	62 (98%)	179 (99%)	194 (97%)	164 (97%)	141 (93%)	499 (96%)	678 (97%)
Carina level^a^									
Detection	58 (100%)	59 (100%)	62 (98%)	179 (99%)	194 (97%)	164 (97%)	141 (93%)	499 (96%)	678 (97%)
Descending aorta									
Detection	58 (100%)	59 (100%)	62 (98%)	179 (99%)	183 (92%)	153 (91%)	134 (89%)	470 (90%)	649 (93%)
Segmentation	58 (100%)	59 (100%)	62 (98%)	179 (99%)	183 (92%)	153 (91%)	134 (89%)	470 (90%)	649 (93%)
Ascending aorta									
Detection	58 (100%)	59 (100%)	62 (98%)	179 (99%)	176 (88%)	143 (85%)	128 (85%)	447 (86%)	626 (90%)
Segmentation	58 (100%)	59 (100%)	62 (98%)	179 (99%)	176 (88%)	143 (85%)	128 (85%)	447 (86%)	626 (90%)
Pulmonary valve^b^									
Detection	58 (100%)	59 (100%)	62 (98%)	179 (99%)	179 (90%)	145 (86%)	129 (85%)	453 (87%)	632 (90%)
Pulmonary trunk									
Detection	58 (100%)	59 (100%)	62 (98%)	179 (99%)	179 (90%)	146 (86%)	130 (86%)	455 (88%)	634 (91%)
Segmentation	57 (98%)	59 (100%)	62 (100%)	179 (99%)	179 (90%)	146 (86%)	130 (86%)	455 (88%)	634 (91%)

Detection—number and percentage of examinations where the anatomical feature was correctly detected. Segmentation—number and percentage of examinations where the anatomical feature was correctly segmented.

^a^A 0.75 cm distance between left main bronchus and right main bronchus was defined as the optimal representation of the carina level.

^b^The pulmonary valve/proximal part of the pulmonary trunk.

### Comparison of measurement performance versus the radiologist

**Figure 4:**
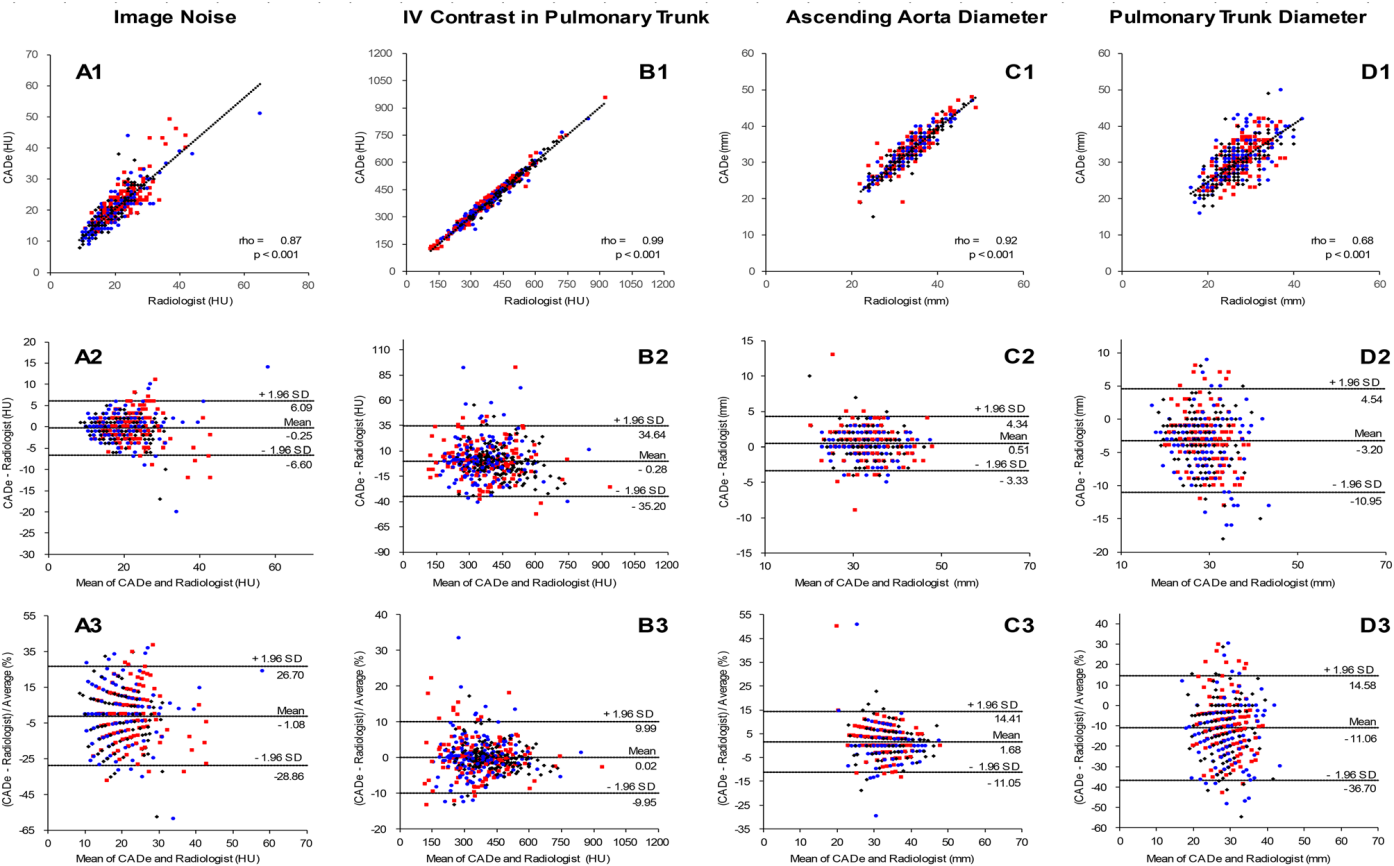
Comparison between the automated CADe system and the radiologist. Automatic and manual measurements from test set cases were compared by regression analysis (top row, dashed regression lines), Bland–Altman plots of differences in radiodensity and diameters (middle row, limits of agreement from − 1.96 to + 1.96 SD) and Bland–Altman plots of percentage differences (bottom row, limits of agreement from − 1.96 to + 1.96 SD). (**A**) Image noise (n = 470 CTPA exams). (**B**) Intravenous contrast agent in PT (n = 455 CTPA exams). (**C**) Ascending aorta diameter (n = 447 CTPA exams). (**D**) Pulmonary trunk diameter (n = 455 CTPA exams). The quality of each CTPA examination was assessed by the radiologist as good (black diamonds), acceptable (blue circles), or inferior (red squares).

Next, we compared the performance of the CADe system versus the radiologist in image noise assessment, IV contrast level measurement in the PT, AAo diameter measurement and PT diameter measurement. The measurements of the CADe system and the radiologist were highly correlated (Pearson’s r = 0.87 for image noise, n = 470; 0.99 for IV contrast in the PT, n = 455; 0.92 for AAo, n = 447; and 0.68 for PT diameter, n = 455) (p < 0.001) (Fig. 4A1–D1). The limits of agreement between the CADe system and the radiologist had mean differences of − 0.25 HU for image noise, 0.28 HU for the IV contrast level measurement in the PT, 0.51 mm for the AAo diameter, and − 3.20 mm for the PT diameter (Fig. 4A2–D2). The Bland–Altman analysis revealed mean differences of − 1.08% for image noise, 0.02% for the IV contrast level measurement in the PT, 1.68% for the AAo diameter, and − 11.06% for the PT diameter. The CTPA image quality slightly affected the measurement performance of the mediastinal vessel structures. The AAo diameter and PT diameter measurements on good quality CTPA examinations showed a stronger correlation between CADe system and manual measurements than those in examinations with inferior image quality (Supplemental Figs. 17–20). The percentage of incorrect CADe measurements greater than ± 1.96 SD in successfully detected compartments was 4% for image noise and AAo diameter, 5% for IV contrast in PT, and 7% for PT diameter (Supplemental Table 2 and Supplemental Fig. 21). While 83% of the AAo diameter measurements were within 0.0 mm and 2.0 mm, only 41% of the PT diameter measurements were within 2.0 mm or less (Supplemental Table 3). When analyzed by examination quality, 85%, 86%, and 76% of the AAo diameter measurements, and 48%, 40%, and 33% of the PT diameter measurements were within the error range of 0.0–2.0 mm for good, acceptable and inferior quality CTPA exams, respectively. The mean deviation between the CADe system and the radiologist was for good, acceptable, and inferior image quality, respectively, 1.39 mm, 1.22 mm, and 1.77 mm for the AAo diameter measurements, and 3.44 mm, 4.32 mm, and 4.15 mm for the PT diameter measurements (Supplemental Fig. 22).

**Figure 5:**
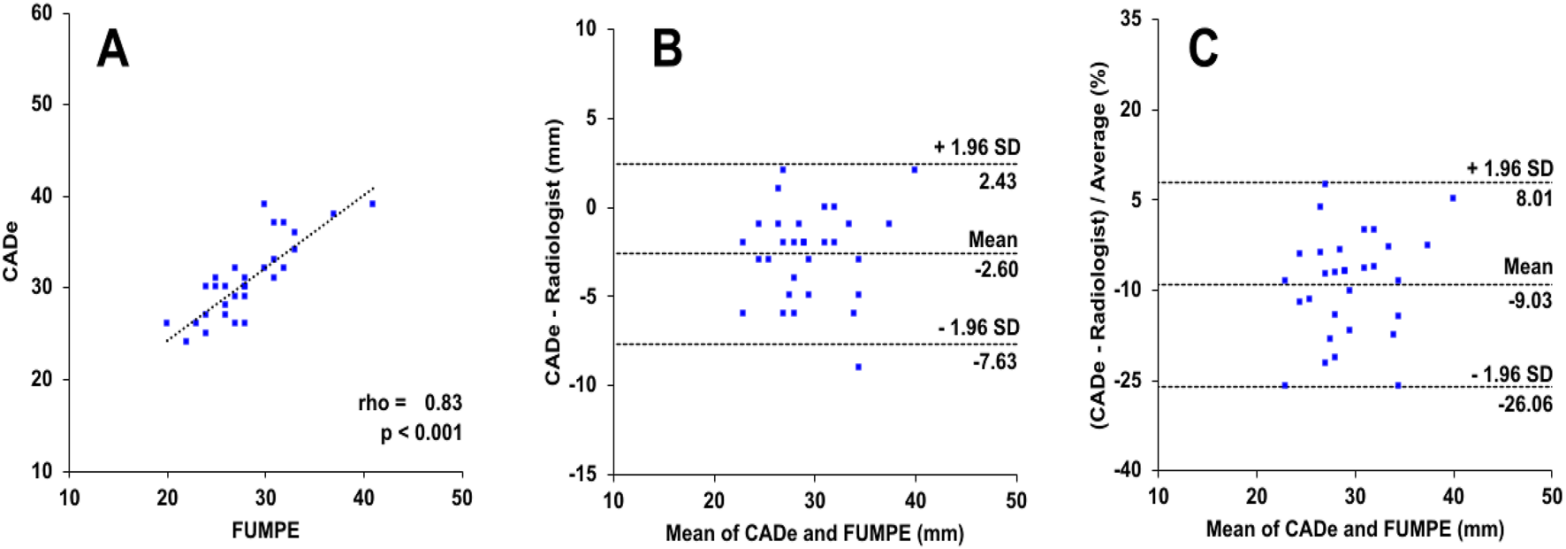
External validation of automatic PT diameter measurement. Automatic PT diameter measurement from the FUMPE dataset cases (n = 31 CTPA exams) was compared to the manual radiologist annotation from the original study by (**A**) regression analysis (dashed regression lines), (**B**) Bland–Altman plots of differences in mm (limits of agreement from − 1.96 to + 1.96 SD) and (**C**) Bland–Altman plots of percentage differences (limits of agreement from − 1.96 to + 1.96 SD).

External validation of the PT diameter measurement was performed in 35 CTPA exams from the FUMPE dataset^23^ where the CADe system successfully measured 31 exams (Pearson’s r = 0.83, p < 0.001) (Fig. 5A). For PT diameter, the limits of agreement between the CADe system and FUMPE radiologist annotation had mean differences of − 2.60 mm and -9.03% (Fig. 5B and C). Thus, the measurements of the automatic system were essentially on par with the radiologist in both our test dataset as well as in an external validation dataset.

### Computational performance of the system

The CADe system was implemented in MATLAB R2019b, and the source code is available at https://github.com/aictpa/GreatVesselsAnalysis. The CADe system was tested on a workstation with Intel Core i9-9900X processors (3.5 GHz). Compartments were determined correctly in 178/180 and 442/520 examinations in the training and test sets respectively, and the computational performance on these examinations is shown in Table 4. The mean number of slices per examination was 146 ± 18 [SD] and the mean uploading time of CTPA examinations to the system was 1.1 ± 0.17 s. The mean run times for detection, segmentation, and measurement of all compartments was 5.4 ± 1 s in serial mode, and 1.2 ± 0.26 s in parallel mode.

**Table 4:** Computational performance of the CADe algorithm on the training and test data sets.

	Quality of CTPA exams	n	Slice numbers	CTPA exam uploading time (s)	Execution time in serial mode (s)^a^	Execution time in parallel mode (s)^b^
Training set	Good	57	142 ± 16	1.1 ± 0.17	5.4 ± 0.84	1.3 ± 0.26
	Acceptable	59	145 ± 16	1.0 ± 0.11	5.4 ± 0.96	1.2 ± 0.30
	Inferior	62	143 ± 15	1.0 ± 0.19	5.5 ± 0.92	1.3 ± 0.27
	All	178	143 ± 16	1.0 ± 0.16	5.4 ± 0.90	1.3 ± 0.27
Test set	Good	175	147 ± 19	1.2 ± 0.17	5.3 ± 1.02	1.2 ± 0.27
	Acceptable	143	148 ± 19	1.2 ± 0.17	5.4 ± 1.01	1.2 ± 0.24
	Inferior	124	144 ± 16	1.1 ± 0.08	5.4 ± 1.04	1.2 ± 0.24
	All	442	147 ± 18	1.1 ± 0.16	5.4 ± 1.02	1.2 ± 0.25
Overall	Good	232	146 ± 19	1.1 ± 0.18	5.3 ± 0.98	1.2 ± 0.27
	Acceptable	202	147 ± 18	1.1 ± 0.16	5.4 ± 1.00	1.2 ± 0.26
	Inferior	186	144 ± 16	1.0 ± 0.15	5.4 ± 1.00	1.2 ± 0.25
	All	620	146 ± 18	1.1 ± 0.17	5.4 ± 1.00	1.2 ± 0.26

Unless otherwise indicated, data are mean ± SD. The running time was calculated as a total for all compartments, excluding data uploading and including detection, segmentation, and measurement tasks per examination. All compartments were determined correctly in 178/180 and 442/520 examinations in the training and test sets respectively. The mean number of slices per examination was 146 ± 18 (SD).

*CTPA* computed tomography pulmonary angiography, *s* second.

^a^Serial mode executes the CTPA examinations sequentially on a single processor.

^b^Parallel mode executes the CTPA examinations in parallel on a multi-processor.

## Discussion

The growing number of examinations performed per radiologist is rapidly becoming a challenge for healthcare^24,25^. Automated systems performing accurate detection, segmentation and measurements in CT imagery could be a solution but have so far not had sufficient performance for clinical implementation. We here developed and tested a deterministic algorithm that automatically detects, segments, and measures mediastinal structures in non- ECG-gated CTPA examinations.

We observed high concordance between the fully automated CADe system and the clinical state-of-the-art in noise assessment and mediastinal vascular measurements. Prior work using traditional image processing techniques to detect the AAo in a smaller set of 90 CTPA examinations had a success rate of 93% with mean absolute difference of AAo diameter measurement between algorithm and radiologist of 1.85 mm^21^. Here, the developed algorithm was 86% successful in detecting the AAo, but the mean absolute difference between algorithm and radiologist was only 0.51 mm. A deep learning based system tested on 288 CTPAs had mean differences between algorithm and radiologist of -0.94 mm for the AAo diameter, and − 0.86 mm for the PT diameter^20^. Another deep learning system to segment multiple cardiovascular structures including AAo, DAo, and PA obtained an overall median Dice score of 0.82 in a validation dataset of 42 examinations^17^. Here, the median Dice score for AAo segmentation was 0.92 in the external validation dataset, which represents a considerable improvement. However, the state-of-the-art methods were tested only in small datasets, whereas our algorithm was tested on a large set of unselected examinations reflecting routine radiology. Altogether, this study advances the fully deterministic detection, segmentation, and measurement of mediastinal structures in CTPA examinations, which is of particular importance for medical device software implementations intended for clinical use.

In clinical practice, a considerable proportion of the CTPA examinations will contain artifacts or other factors that reduce examination quality which complicates manual as well as automatic analysis. However, the quality of examinations has not been in focus in previous CTPA CADe work^17,18,21^. The high median age of patients under investigation for PE entails a higher prevalence of age-related anatomical deviations in the chest and mediastinum; it is therefore essential to train CADe systems on unselected examinations that reflect this clinical reality. Here, the system performed slightly better on CTPA examinations with good image quality than those with acceptable and inferior image qualities. Interestingly, system performance was only marginally worse for detection of anatomical landmark and cardiovascular structures in the examinations deemed of inferior quality as compared to those of acceptable quality. Examination quality had most impact on PT segmentation, mainly due to streak artifacts from the superior vena cava. High IV contrast within mediastinal structures may cause streak artifacts, which can alter the appearance of adjacent organs and make it difficult for the CADe system to detect and segment the organs. Examination quality also had an impact on the accuracy of diameter measurements. Differences in scanner models and CTPA protocols could potentially impact CADe performance if it was implemented at another hospital. Here, the CADe consistency is supported by the high similarity between results when the external SegTHOR and FUMPE datasets were used to validate different aspects of the system. Taken together, the developed system performs well in internal as well as external datasets independently of examination quality.

A limitation of the system is that the order of segmentation of anatomical structures is sequential. As the DAo was found to be relatively homogeneous and easily detected by a computer, DAo segmentation was performed before AAo and PT segmentation. The primary advantage of this approach is full automation without any input from the radiologist, but the main disadvantage is that failure at any step in the segmentation chain affects downstream detection and segmentation.

Unlike probabilistic approaches, such as deep convolutional neural networks (DCNN), the proposed deterministic model does not necessitate pixel-wise annotations for segmentation tasks. Therefore, the primary contribution of this approach is the elimination of the need for expert-level annotations during model development. Additionally, unlike the black box problem present in probabilistic approaches, all segmentation and measurement errors or unexpected outcomes can be easily tracked due to the inherent transparency of deterministic systems. In future work, several aspects need to be addressed. First, to fully leverage the proposed CADe system, the results should be integrated into the PACS server. One of the simplest means for achieving this is by generating Grey Scale Presentation State (GSPS) objects for segmentation and measurement outputs. This would enable the analysis of results generated by GSPS objects using any universal image viewer available on the PACS server. Second, there are additional structures that can provide prognostic information or serve as diagnostic indicators, such as volumetric analysis of the heart chambers, the right to left ventricle diameter ratio, or contrast reflux into the inferior vena cava^26^. Third, significant abnormalities may occur in structures at other anatomical levels. These anomalies in neighboring sections of the structure of interest can be identified by creating a 3D segmentation mask through the training of a deep learning model, such as the 3D U-Net model. To generate a 2D mask for training the deep learning model the segmentation output from the proposed CADe system can be utilized. Finally, our internal dataset with its corresponding annotations comprises solely CTPA volumes. Nonetheless, it is worth noting that the system was capable of processing contrast-enhanced CT images from the SegTHOR dataset. Therefore, as part of our future work, we intend to expand our testing to a larger set of contrast-enhanced CT images.

To the best of our knowledge, this system represents the first CTPA algorithm developed using a large number of cases with an adequate representation of common artifacts and challenges encountered in clinical radiology. The automatic CADe system detected and segmented anatomical landmarks, measured vascular structures of interest and determined relevant parameters of image quality. With its excellent computational performance, the system enables automatic assessment of large image volumes. This could also be used for research to clarify the diagnostic and prognostic impact of morphological and geometric properties of mediastinal structures. The algorithm can serve as a foundation for further CT chest AI development and already in its current form provide radiologists with valuable automated measurements.

## Materials and methods

### Dataset

The dataset consists of 700 retrospective non-electrocardiogram (ECG)-gated CTPA examinations performed at a single institution (Nyköping Hospital, Nyköping, Sweden) between 2014 and 2018; 383 CTPA examinations from 353 women (age range 16–97; median age 73; interquartile range 20) and 317 from 299 men (age range 19–100; median age 71; interquartile range 15). The CTPAs were performed in four different time periods to include examinations from several different CT scanners. Consecutive CTPAs ensured an image material with adequate representation of gender, age, common artifacts and medical conditions. The examinations were randomly assigned to a training set (n = 180) used for algorithm development, and a test set (n = 520) for unbiased evaluation of the final algorithm. Collection and analysis of CTPA examinations was approved by the Swedish Ethical Review Authority (EPN Uppsala Dnr 2015/023 and 2015/023/1). All personal identifiers in Digital Imaging and Communications in Medicine (DICOM) headers were removed from the dataset (Dicom2usb). We validated the proposed computer-aided detection (CADe) system on two publicly available datasets: 12 CTPAs from Segmentation of Thoracic Organs at Risk (SegTHOR^22^) (AAo segmentation), and 35 CTPA exams from the Ferdowsi University of Mashhad’s PE (FUMPE^23^) dataset (PT diameter measurement).

### CT image acquisition

The non-ECG-gated CTPA examinations were conducted with 5 different multidetector-row CT scanners (Brilliance 64, Ingenuity Core and Ingenuity CT, Philips Medical Systems; LightSpeed VCT, General Electric (GE) Healthcare Systems; Somatom Definition Flash, Siemens Healthcare) after IV injection of contrast (Omnipaque 350 mg I/ml, GE Healthcare Systems) and saline. The CT image acquisition technique varied by manufacturer with most frequent slice thickness of 0.625 mm (0.625 mm–2.0 mm), pixel spacing of 0.7 mm (0.59 mm–0.98 mm), and voltage of 100 kV (80 kV–120 kV). A secondary axial reformat with 2.0 mm slice thickness was performed on all examinations.

### Measurements and image quality assessment by the radiologist

**Figure 6:**
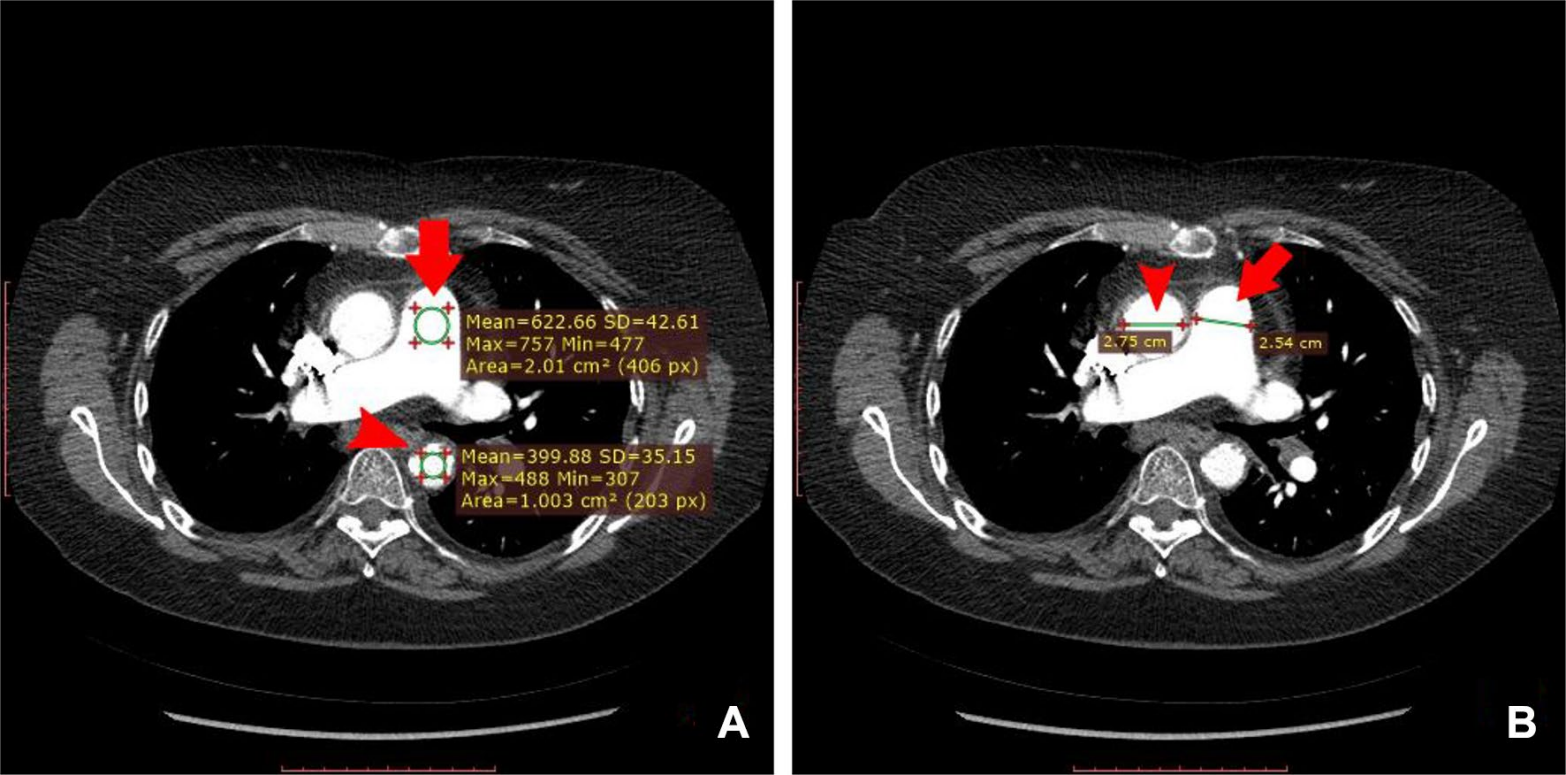
Ground truth measurements by the radiologist. The measurements were performed in the axial 2 mm image which optimally presented the PT. (**A**) The IV contrast concentration in the PT was recorded as the mean HU value in a 2 cm^2^ circular region of interest (arrow). Image noise was determined as the SD of HU in a 1 cm^2^ circular region of interest in the DAo (arrowhead). (**B**). The diameters of the PT (arrow) and AAo (arrowhead) were measured.

**Figure 7:**
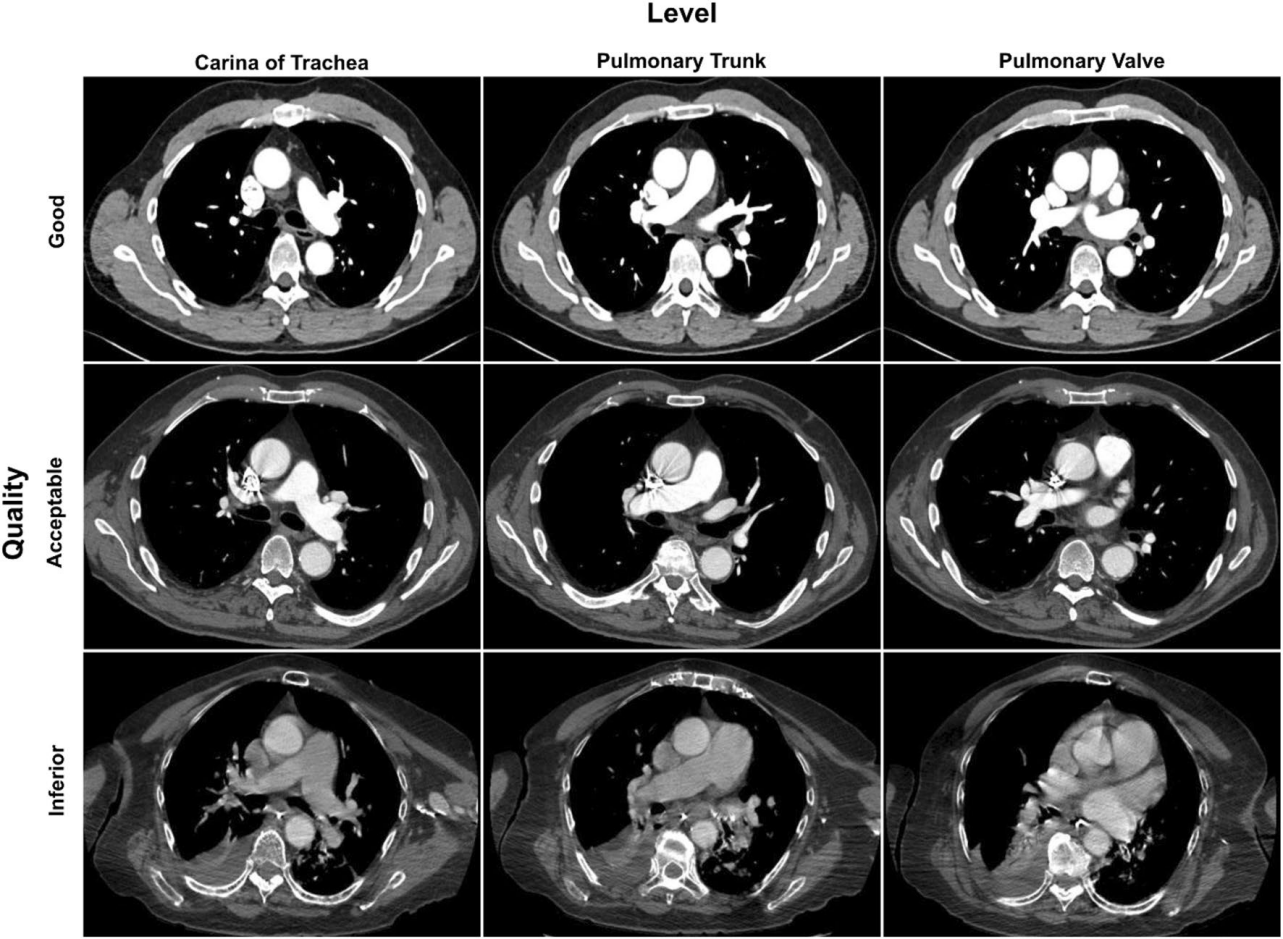
Representative examples of CTPA examinations of different image quality. The examinations were classified by the radiologist as being of good, acceptable or inferior quality. The levels of the carina of the trachea, the PT and the PV are shown with the same window setting (width = 600 HU, level = 100 HU).

The CTPA examinations were exported from the Picture Archiving and Communication System (PACS, Sectra AB) in DICOM format. The examinations were reviewed and annotated using the RadiAnt DICOM Viewer (Medixant) by a senior radiologist (TF) with 15 years of experience. Of the CTPAs, 150 were first reviewed and annotated by a radiology resident (DT) with 5 years of experience in general diagnostic radiology and then double read by TF. In the 2 mm axial image stack, the image which optimally presented the PT was identified and the diameter of the PT and AAo, IV contrast concentration in PT (mean value of HU in 2 cm^2^ circular ROI), and image noise (SD of HU in a 1 cm^2^ circular ROI in the DAo) were measured by the radiologist and used as ground truth (Fig. 6). For each CTPA examination the radiologist also scored five image quality parameters affecting the evaluation for PE: motion artifacts, streak artifacts, IV contrast concentration in PT, parenchymal disease, and image noise (Supplemental Methods: Image score calculations and Supplemental Table 1). A total score was calculated, and the result determined the overall CTPA examination quality as good (score 0–3), acceptable (4–7) or inferior (≥ 8) (Fig. 7).

### CADe system

To automatically detect, segment and measure mediastinal structures (AAo, DAo, PT), we combined image processing and image analysis techniques to detect and segment compartments. The developed CADe system does not require any user interaction and consists of two main steps, pre-processing and a segmentation chain (Fig. 1 and Supplemental Fig. 1).

### Pre-processing

For each axial CT image, every voxel was converted to HU and the direction of scanning was determined based on information in the DICOM header. As patient orientation may vary along the cranial to caudal direction because of scoliosis, movement or position during the examination, or other reasons, the CT exam was aligned with respect to the x-axis of the axial plane in the cranial as well as caudal part of the examination (Fig. 1B1 and B2, Supplemental methods: Description of the CADe system 1–4, and Supplemental Figs. 2–6).

### Segmentation

First, the system located the three-dimensional positions of the carina of trachea and an apical level of the pulmonary valve (PV), to find seed points of vascular structures in the mediastinum. Second, the seed points for segmentation were automatically placed using a heuristic approach. Third, the structures were segmented by applying image processing techniques for image enhancement, edge detection, gray scale segmentation, and 2D region growing. Finally, measurements were done on the segmented regions (Supplemental Figs. 7–14).

#### Locating the trachea and airways

Details are found in Supplemental methods: Description of the CADe system 5–8. The CADe system extracts two adjoined volumes of interest from the superior part of the thoracic cavity, wherein trachea candidates are generated and assessed separately (Fig. 1C4 and C5). In these two volumes, three air-filled structures (the trachea and the left and right lung) are identified by thresholding, flood-fill operations and connected component analyses. Trachea candidates are then joined across the two volumes and connected component analyses are applied. The longest of the candidates also having diameter and volume within an empirically determined range is selected as the trachea. When the trachea has been detected, it is tracked cranially to caudally slice by slice to the bifurcation point where the left and right main bronchi can be found as two distinct segments (Fig. 1C6). We then designate the CT slice, where the distance between the left and right main bronchus is > 0.75 cm, as the level of the carina of the trachea. Thus, the trachea and the carina of the trachea can be located automatically in 3D.

#### Locating and measuring vascular structures of the mediastinum

Details are found in Supplemental methods: Description of the CADe system 9–16. We observed that the DAo can be easily detected around the level of carina trachea since the DAo is always located posterior to the left main bronchus and its appearance (IV contrast concentration and circularity) is homogeneous around the level of carina trachea. Two sequential sets of artificial ray search spaces were made. One or more rays passing a sufficient number of connected pixels to correspond to the contrast filled aorta indicate that the DAo has been located (Fig. 1C9). Once the DAo was detected, a segmentation chain was applied to detect the aortic arch. The area of a 1 cm^2^ circle at the mass center of the DAo was used to calculate the average HU density of the CTPA examination as a metric of contrast filling (Fig. 1C12). The AAo can be detected by tracking and comparing the segmented DAo regions slice by slice in the caudal to cranial direction to find features characteristic of the AAo. Morphological changes indicate the level of the aortic arch (Fig. 1C13). By tracking the anterior part of the aortic arch in the cranial to caudal direction slice by slice the first identified circular object was designated as AAo (Fig. 1C14). Next, we segmented the AAo between the levels of the aortic arch and the carina of trachea (Fig. 1C15) and calculated the diameter of AAo as the mean of AAo diameters in these planes. The PT is adjacent to the AAo and remains on its left. We therefore created a rectangular search space adjacent to the left lateral side of AAo (Fig. 1C18) and applied our segmentation pipeline to this search space to segment the PT. Next, the PT was tracked in the cranial-to-caudal direction to reach the PV/proximal part of the PT. The circularity of the segmented region was used to determine whether the level of the PV had been reached (Fig. 1C20). The tracked PT diameter was calculated by Hough transform (Fig. 1C20) as the mean of PT diameters in these planes. Taken together, the DAo, AAo and PT were automatically detected in 3D and their average diameters and contrast levels obtained.

### Statistical analysis

Statistical analysis was performed using Microsoft Office Excel (Microsoft Corporation, Office Professional Plus 2016). Bland–Altman and scatter analyses were used to compare agreement and relationship, respectively, between automated and manual measurements. A p-value of < 0.05 was defined as statistically significant and Pearson’s correlation coefficient was used to evaluate the agreement between automated measurement and manual measurement. The accuracy of the CADe system was assessed by Boundary F1 score and the Dice coefficient score using Matlab (MathWorks, Inc., R2019b).

### Supplementary Information

Supplementary Information.

## Data Availability

The datasets generated during and/or analysed during the current study are available from the corresponding author on reasonable request.

## Code availability

The developed model code can be found at the following link. https://github.com/aictpa/GreatVesselsAnalysis.
