# Supplementary material for: Automated detection, segmentation and measurement of major vessels and the trachea in CT pulmonary angiography
Source: Sci Rep. 2023 Oct 27;13:18407. doi: 10.1038/s41598-023-45509-1 (PMC10611811; doi:10.1038/s41598-023-45509-1)
Supplement: Supplementary file 1 — Supplementary Information. [file 41598_2023_45509_MOESM1_ESM.docx]

**Supplemental Information**

**Automated Detection, Segmentation and Measurement of Mediastinal Structures in CT Pulmonary Angiography**

Ali Teymur Kahraman, Tomas Fröding, Dimitrios Toumpanakis, Natasa Sladoje, Tobias Sjöblom.

**Supplemental Tables**

Supplemental Table 1 Parameters for calculation of image quality as assessed by the radiologist

Supplemental Table 2 Frequency of CADe measurements of successfully detected compartments deviating more than ± 1.96 SD from the radiologist.

Supplemental Table 3 Percentage of CADe measurements with various deviation ranges from the radiologist’s measurement (mm)

**Supplemental Figures**

Supplemental Figure 1. Flowchart of the developed system.

Supplemental Figure 2. Orientation of the CT slice.

Supplemental Figure 3. Algorithmic steps of patient orientation calculation method I.

Supplemental Figure 4. Accurate orientation of the CT scan.

Supplemental Figure 5. Algorithmic steps of patient orientation calculation method II.

Supplemental Figure 6. Visualization of the patient orientation with respect to the x-axis.

Supplemental Figure 7. Flowchart of the descending aorta detection.

Supplemental Figure 8. Placement of artificial rays for descending aorta detection.

Supplemental Figure 9. Calculated edges and borders of the tissues in a CT slice.

Supplemental Figure 10. Visualization of the region of interest for the descending aorta searching.

Supplemental Figure 11. Tracking the anterior part of the aortic arch.

Supplemental Figure 12. The search space for the pulmonary trunk.

Supplemental Figure 13. Tracking area for the pulmonary trunk.

Supplemental Figure 14. Placement of the Hough transform rays into pulmonary trunk.

Supplemental Figure 15. Representative results of the graphical output of the CADe system.

Supplemental Figure 16. Fully automated detection of anatomical landmarks and vascular structures in CTPA examinations of good, acceptable or inferior image quality.

Supplemental Figure 17. Image quality dependence of noise assessment.

Supplemental Figure 18. High performance in intravenous contrast agent measurement in the pulmonary trunk independent of image quality.

Supplemental Figure 19. Image quality dependent performance in ascending aorta diameter measurements.

Supplemental Figure 20. Image quality dependent performance in pulmonary trunk diameter measurements.

Supplemental Figure 21. Examples of incorrect CADe measurements on correctly detected mediastinal structures.

Supplemental Figure 22. Image quality dependent diameter measurement deviation between the CADe system and the radiologist.

Supplemental Figure 23. An example of a blank image.

Supplemental Figure 24. Examples of successful detection of the compartments.

Supplemental Figure 25. Examples of successful segmentation of the compartments.

**Supplemental Methods**

Image quality score calculation

Description of the CADe system

Evaluation Criteria for the Detection Tasks

Evaluation Criteria for the Segmentation Tasks

**Supplemental Table 1 Parameters for calculation of image quality as assessed by the radiologist**

| **Aspect** | **Weighting Factor (k)** | **Point-Scale (ps)** | | | | |
| --- | --- | --- | --- | --- | --- | --- |
|  |  | **Conspicuity** | | | **Score** | |
| Motion-Breathing Artifacts (mba) | 4 | Mild | | | 0 | |
|  |  | Moderate | | | 1 | |
|  |  | Severe | | | 2 | |
|  |  |  | | |  | |
| Streak Artifact^a^ (sa) | 1 | Mild | | | 0 | |
|  |  | Moderate | | | 1 | |
|  |  | Severe | | | 2 | |
|  |  |  | | |  | |
| Image Noise^b^ (n) | 1 | *Slice Thickness = 2.0 mm* | |  | | |
|  |  | ≤ 25 | | 0 | | |
|  |  | 26-50 | | 1 | | |
|  |  | 51-100 | | 2 | | |
|  |  | >100 | | 100 | | |
|  |  |  |  |  | | |
| Contrast Concentration in PT^c^ (d) | 1 | > 350 | | | 0 | |
|  |  | 301-350 | | | 1 | |
|  |  | 251-300 | | | 2 | |
|  |  | 201-250 | | | 3 | |
|  |  | 151-200 | | | 4 | |
|  |  | 101-150 | | | 8 | |
|  |  | ≤ 100 | | | 100 | |
|  |  |  | | |  | |
| Lung Disease^d^ (ld) | 2 | No | | | 0 | |
|  |  | Yes | | | 1 | |
| PA = pulmonary artery, PE = pulmonary embolism, PT = pulmonary trunk. | | | | | |  |
| ^a^ Streak artifact from superior vena cava affecting the evaluation of the PAs for PE. | | | | | |  |
| ^b^ SD of Hounsfield units in a circular region of interest of 1 cm² in the descending aorta at the level of PT, measured in 2 mm axial image. | | | | | |  |
| ^c^ Average Hounsfield units in a circular region of interest of 2 cm² in PT, just proximal to the bifurcation. | | | | | |  |
| ^d^ Lung parenchymal disease adjacent to PAs affecting the evaluation for PE. | | | | | |  |

**Supplemental Table 2 Frequency of CADe measurements of successfully detected compartments deviating more than ± 1.96 SD from the radiologist.**

|  |  | Quality of CTPA Exams | | |  |
| --- | --- | --- | --- | --- | --- |
| Measurement |  | Good | Acceptable | Inferior | All |
| Image Noise |  | 3/183 (1.64%) | 7/153 (4.58%) | 9/134 (6.72%) | 19/470 (4.04%) |
| IV Contrast in Pulmonary Trunk |  | 4/179 (2.23%) | 11/146 (7.53%) | 8/130 (6.15%) | 23/455 (5.05%) |
| Ascending Aorta Diameter |  | 5/176 (2.84%) | 4/143 (2.80%) | 9/128 (7.03%) | 18/447 (4.02%) |
| Pulmonary Trunk Diameter |  | 7/179 (3.91%) | 15/146 (10.27%) | 9/130 (6.92%) | 31/455 (6.81%) |

**Supplemental Table 3 Percentage of CADe measurements with various deviation ranges from the radiologist’s measurement**

| Measurement | Quality of CTPA Exams |  | | | | | | | | |  |
| --- | --- | --- | --- | --- | --- | --- | --- | --- | --- | --- | --- |
|  |  |  |  |  |  |  |  |  |  |  |  |
|  |  | Deviation range from the radiologist’s measurement (mm) | | | | | | | | |  |
|  |  |  |  |  |  |  |  |  |  |  |  |
|  |  | **0** | **1** | **2** | **3** | **4** | **5** | **6 - 9** | **10 - 12** | **13 - 18** |  |
| Ascending aorta diameter | Good (n=176) | 38 (21.6) | 77 (43.7) | 35 (19.9) | 17 (9.6) | 6 (3.4) | 1 (0.6) | 1 (0.6) | 1 (0.6) | 0 (0.0) |  |
|  | Acceptable (n=143) | 42 (29.4) | 55 (38.4) | 26 (18.2) | 13 (9.1) | 6 (4.2) | 1 (0.7) | 0 (0.0) | 0 (0.0) | 0 (0.0) |  |
|  | Inferior (n=128) | 21 (16.4) | 50 (39.1) | 26 (20.3) | 16 (12.5) | 10 (7.8) | 3 (2.3) | 1 (0.8) | 0 (0.0) | 1 (0.8) |  |
|  | All (n=447) | 101 (22.6) | 182 (40.7) | 87 (19.5) | 46 (10.3) | 22 (4.9) | 5 (1.1) | 2 (0.5) | 1 (0.2) | 1 (0.2) |  |
|  |  |  |  |  |  |  |  |  |  |  |  |
| Pulmonary trunk diameter | Good (n=179) | 21 (11.7) | 32 (17.9) | 33 (18.4) | 24 (13.4) | 23 (12.9) | 11 (6.2) | 26 (14.5) | 6 (3.3) | 3 (1.7) |  |
|  | Acceptable (n=146) | 8 (5.5) | 26 (17.8) | 24 (16.4) | 22 (15.1) | 7 (4.8) | 15 (10.3) | 32 (21.9) | 5 (3.4) | 7 (4.8) |  |
|  | Inferior (n=130) | 3 (2.3) | 22 (16.9) | 18 (13.8) | 26 (20.0) | 17 (13.1) | 7 (5.4) | 29 (22.3) | 7 (5.4) | 1 (0.8) |  |
|  | All (n=455) | 32 (7.0) | 80 (17.6) | 75 (16.5) | 72 (15.8) | 47 (10.4) | 33 (7.3) | 87 (19.0) | 18 (4.0) | 11 (2.4) |  |
| For each deviation range, the number of examinations and the percentage of examinations (in parentheses) are shown for each specified quality. CTPA = computed tomography pulmonary angiography. | | | | | | | | | | | |
|  | | | | | | | | | | | |

**Supplemental Figure 1. Flowchart of the developed system.**

| 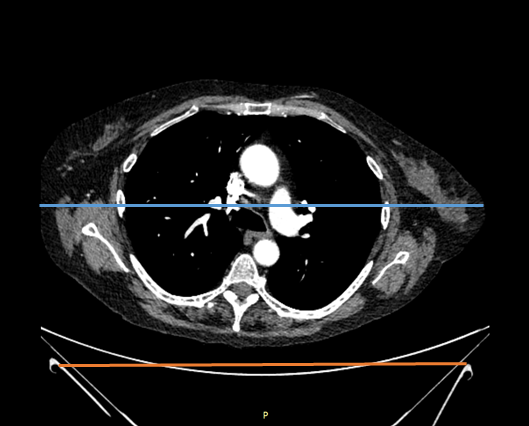 |  | 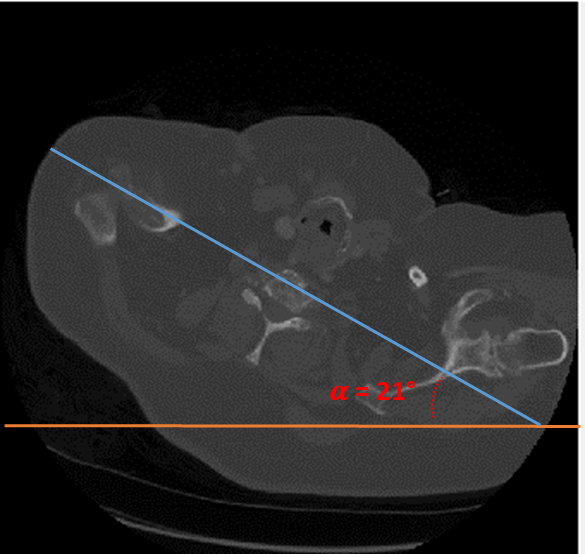 |
| --- | --- | --- |

(a) (b)


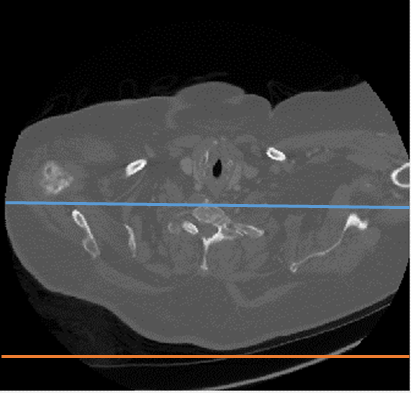


(c)

**Supplemental Figure 2. Orientation of the CT slice.** The blue line represents the major axis of the patient and the orange line represents the major axis (x-axis) of the image. (a) Both lines are parallel to each other. Therefore, the angle between the blue and orange lines (the orientation of the CT slice) is near 0, which is required for proper functioning of the automated system. In (b) the angle between the blue and the orange line is 21 degrees. In order to detect anatomical landmarks (carina of trachea, level of the pulmonary valve) and major mediastinal structures (ascending and descending aorta, pulmonary trunk) accurately, the major axis of the patient must be rotated 21 degrees respect to the major axis of image. The rotated CT scan is represented in (c).

**Supplemental Figure 3. Algorithmic steps of patient orientation calculation method I.**


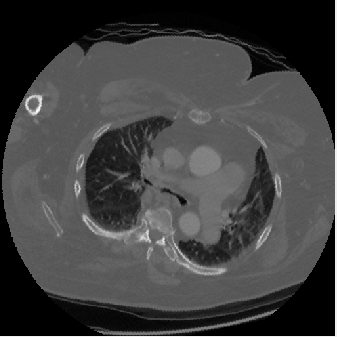

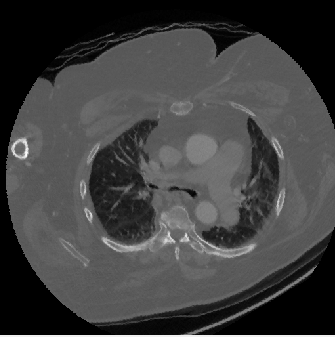


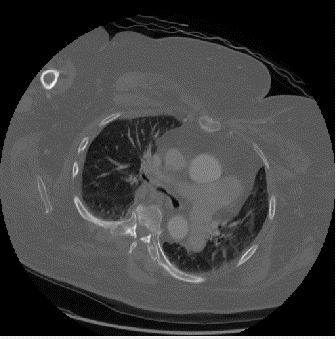


**𝜶 = 27°**

(a) (b) (c)

**Supplemental Figure 4. Accurate orientation of the CT scan.** The orientation of the original image in (a) is 27°. Thus, the image should be rotated 27 degrees to be parallel with respect to the x-axis. If the orientation is calculated with the method proposed in step 3, the result is 12° and the rotated CT scan can be seen in (b). The incomplete rotation interferes with automatic detection of the carina of trachea and the pulmonary trunk. However, with the method proposed in Supplemental Methods, Description of the CADe system, step 4, the calculated orientation is 30° (c) which is sufficiently accurate for proper functioning of the developed system.

**Supplemental Figure 5. Algorithmic steps of the patient orientation calculation method II.**


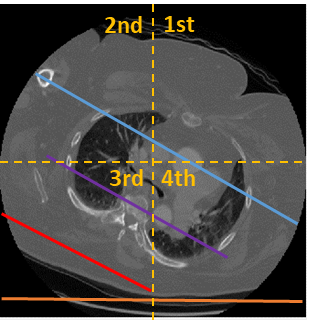


**Supplemental Figure 6. Visualization of the patient orientation with respect to the x-axis.** The solid lines represent the major axis of the patient (blue), the major axis (x-axis) of the image (orange), the major axis of the spine (purple) and the best fitted line of boundary of region 3 (red). The dashed yellow lines delineate quadrants of CT scans. The blue and purple and red lines are parallel to each other. Therefore, it is sufficient to calculate the angle of the red line with respect to the image x-axis to know the orientation of the patient.

**Supplemental Figure 7. Flowchart of the descending aorta detection.**


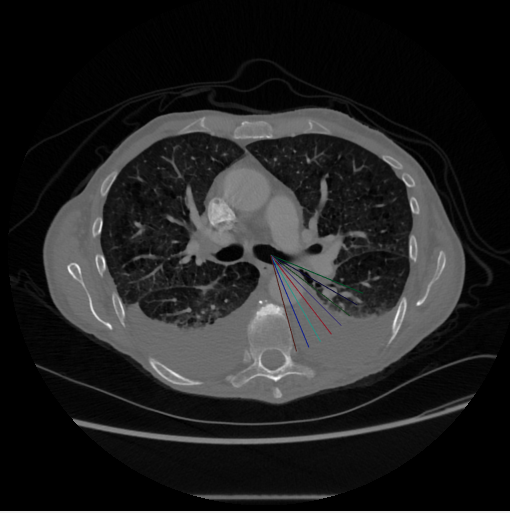

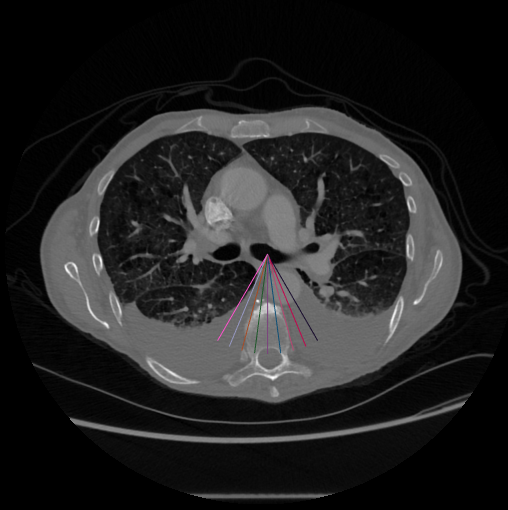


**Supplemental Figure 8.** **Placement of artificial rays for descending aorta detection.** Artificial rays defining search space 1 (the left image) and search space 2 (the right image) are shown in color.


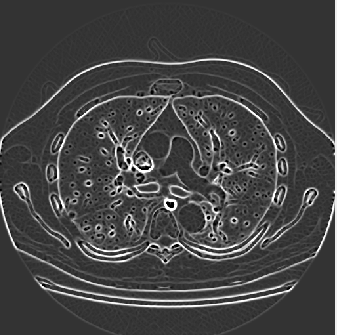

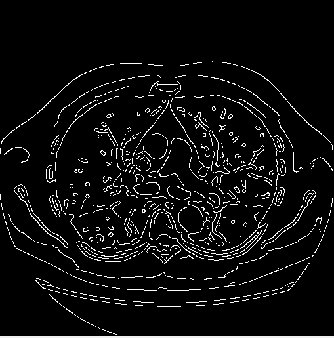


(a) (b)


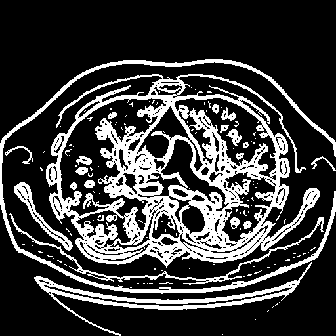


(c)

**Supplemental Figure 9. Calculated edges and borders of the tissues in a CT slice.** The Eigen value ($\lambda_{1}$) of hessian matrix ($H$) is illustrated in (a), the results of the Canny edge detection operation in (b), and the combination in (c).


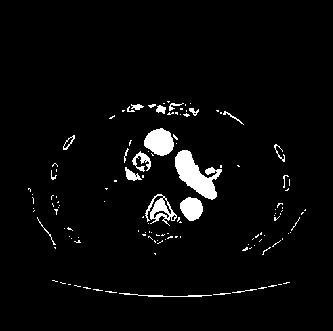


**Supplemental Figure 10. Visualization of the region of interest for the descending aorta searching.** The region of interest is shown in white.


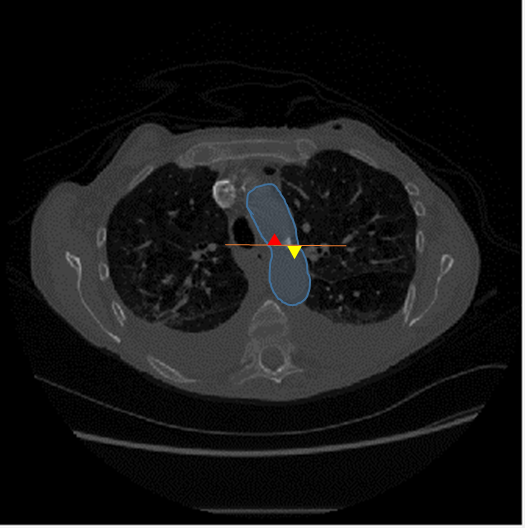


**Supplemental Figure 11. Tracking the anterior part of the aortic arch.** The area delineated by blue line is the aortic arch in one CT slice. The orange line is an artificial line, which divides the aortic arch into two parts; the anterior part (indicated by red triangle) and the posterior part (indicated by yellow triangle).


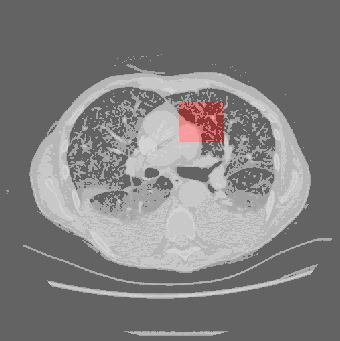


**Supplemental Figure 12. The search space for the pulmonary trunk.** The area delineated by the red rectangle is the search area for the PT in the CT slice.


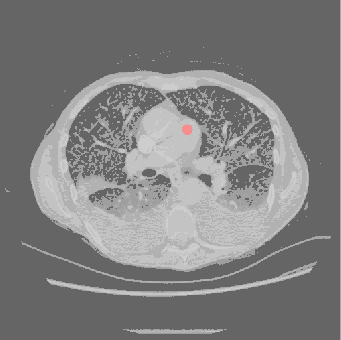


**Supplemental Figure 13. Tracking area for the pulmonary trunk.** The area delineated by the red circle is the sampling area for tracking the PT to the level of the PV in the cranial to caudal direction.


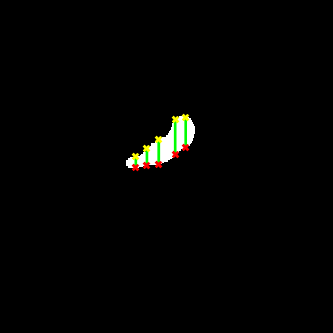

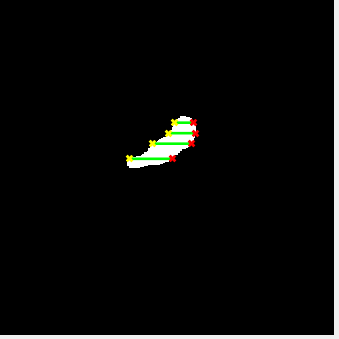


**Supplemental Figure 14.** **Placement of the Hough transform rays into the pulmonary trunk.** The horizontal (image on the left) and the vertical (image on the right) lines are found by applying the Hough transform and are then calculated.

**
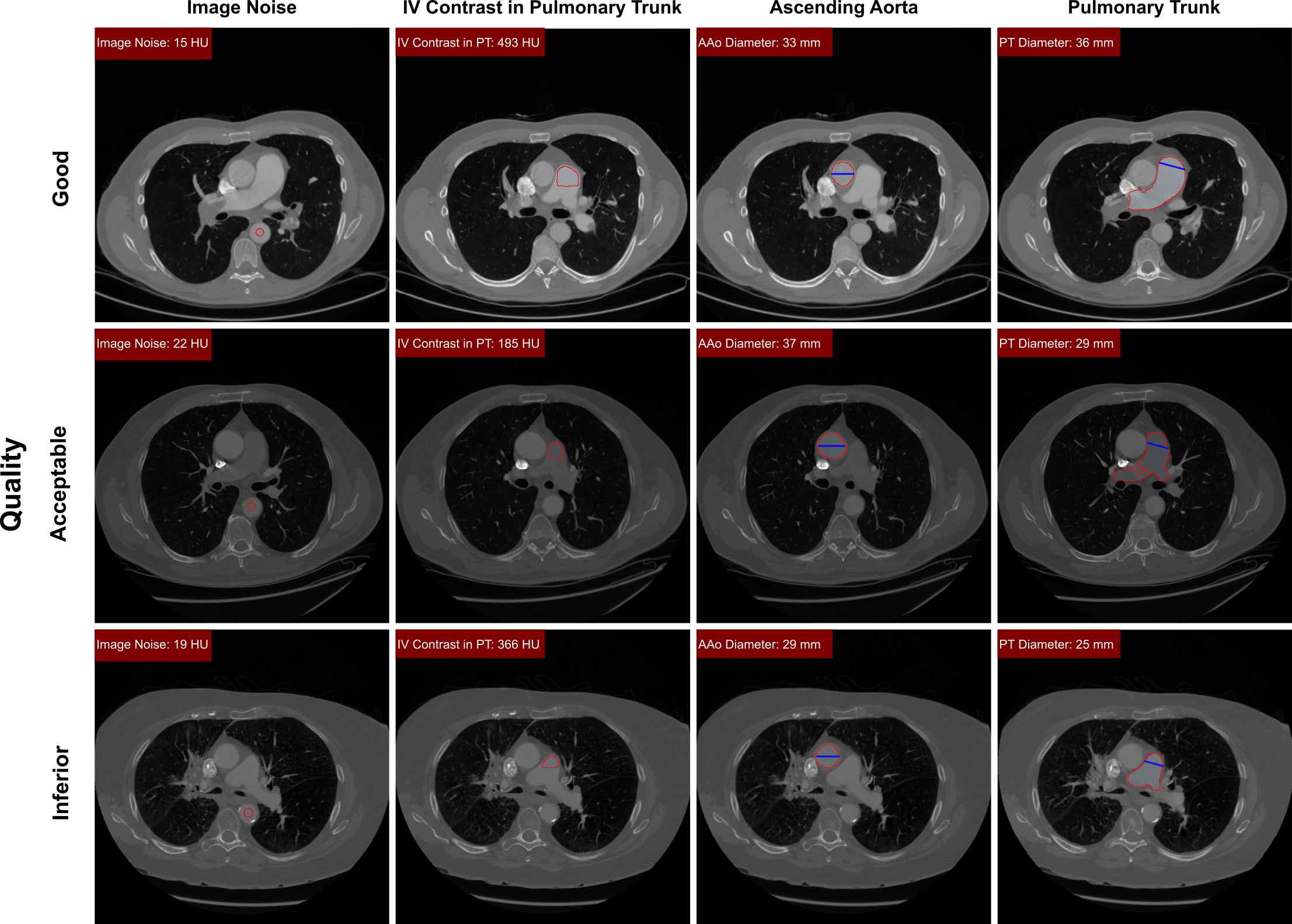
**

**Supplemental Figure 15.** **Representative results of the graphical output of the CADe system.** Noise assessment and measurements of mediastinal vascular structures are shown. Red line, regions of interest for measurements; blue line, CADe diameter measurement; top left, numerical values of CADe measurements. Examinations of good, acceptable and inferior quality are shown for comparison. The radiologist’s manual measurements are given for image noise (Good, 16 HU; Acceptable, 19 HU; Inferior, 20 HU), IV contrast in PT (Good, 493 HU; Acceptable, 175 HU; Inferior, 332 HU), AAo diameter (Good, 34mm; Acceptable, 36 mm; Inferior, 32 mm), and PT diameter (Good, 36 mm; Acceptable, 29 mm; Inferior, 25 mm).


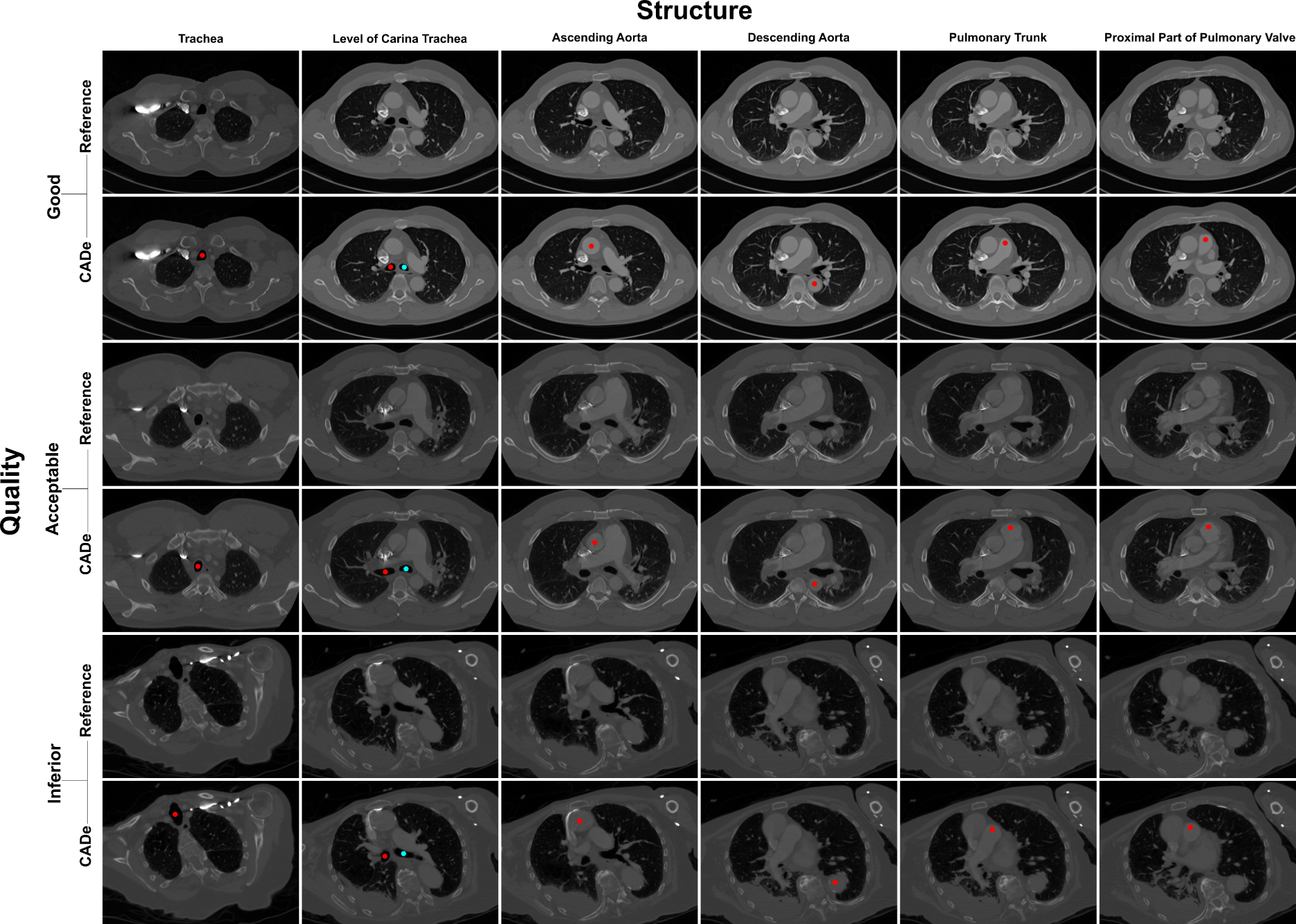


**Supplemental Figure 16.** **Fully automated** **detection of anatomical landmarks and vascular structures in CTPA examinations of good, acceptable or inferior image quality.** The CADe system places a dot on the detected compartments. For the illustration, the detection results of the software are indicated by red (right main bronchus) and aqua (left main bronchus) circles.


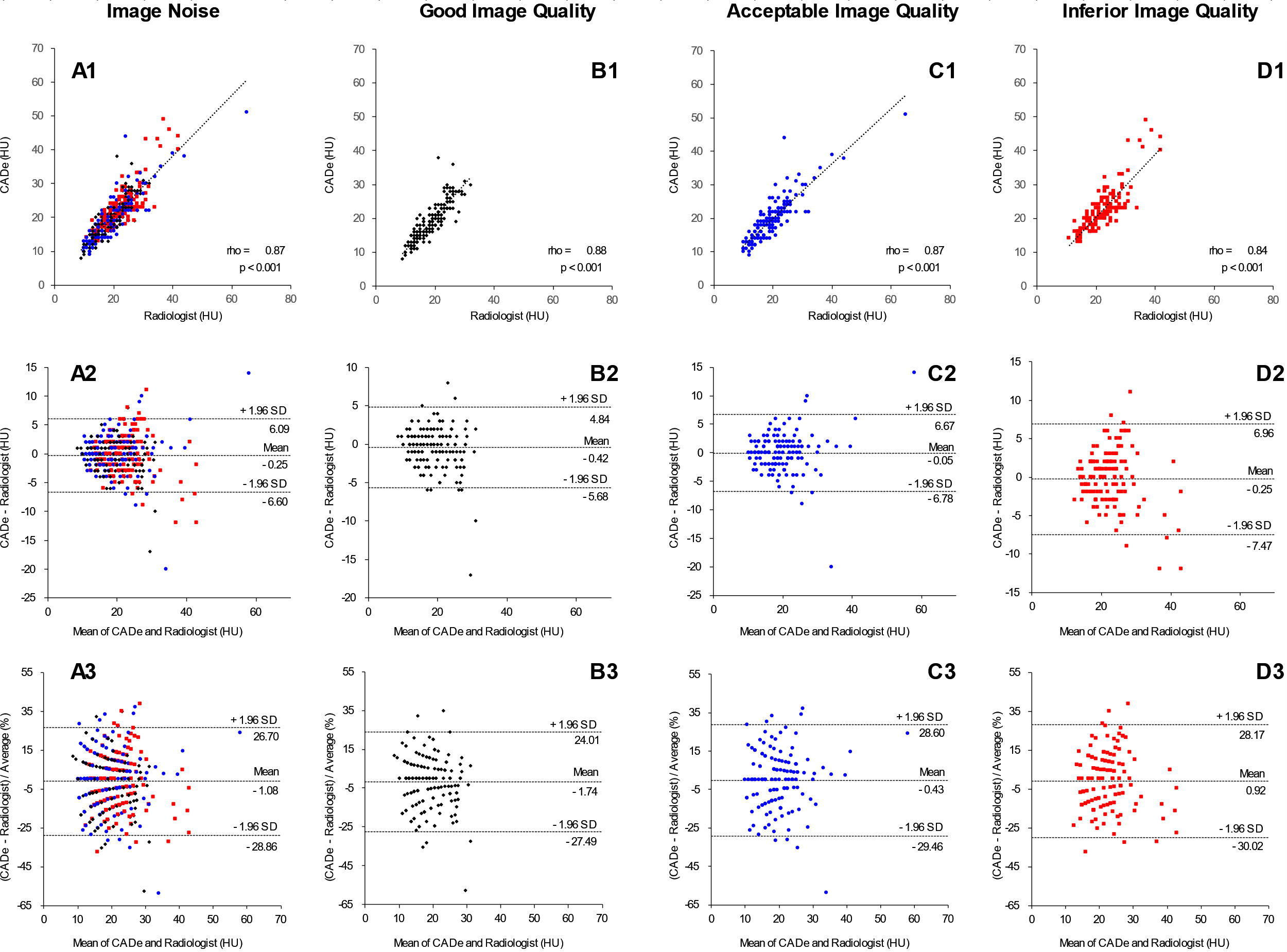


**Supplemental Figure 17. Image quality dependence of noise assessment.** Automatic and manual measurements from test set cases were compared by regression analysis (top row, dashed regression lines), and Bland-Altman plots of differences in HU (middle row, limits of agreement from -1.96 to +1.96 SD) and Bland-Altman plots of differences in percentage (bottom row, limits of agreement from -1.96 to +1.96 SD). A. Images of all qualities (n=470 CTPA exams). B. Good image quality (n=183 CTPA exams). C. Acceptable image quality (n=153 CTPA exams). D. Inferior image quality (n=134 CTPA exams). The quality of the CTPA examinations was assessed by the radiologist as good (black diamonds), acceptable (blue circles), or inferior (red squares).


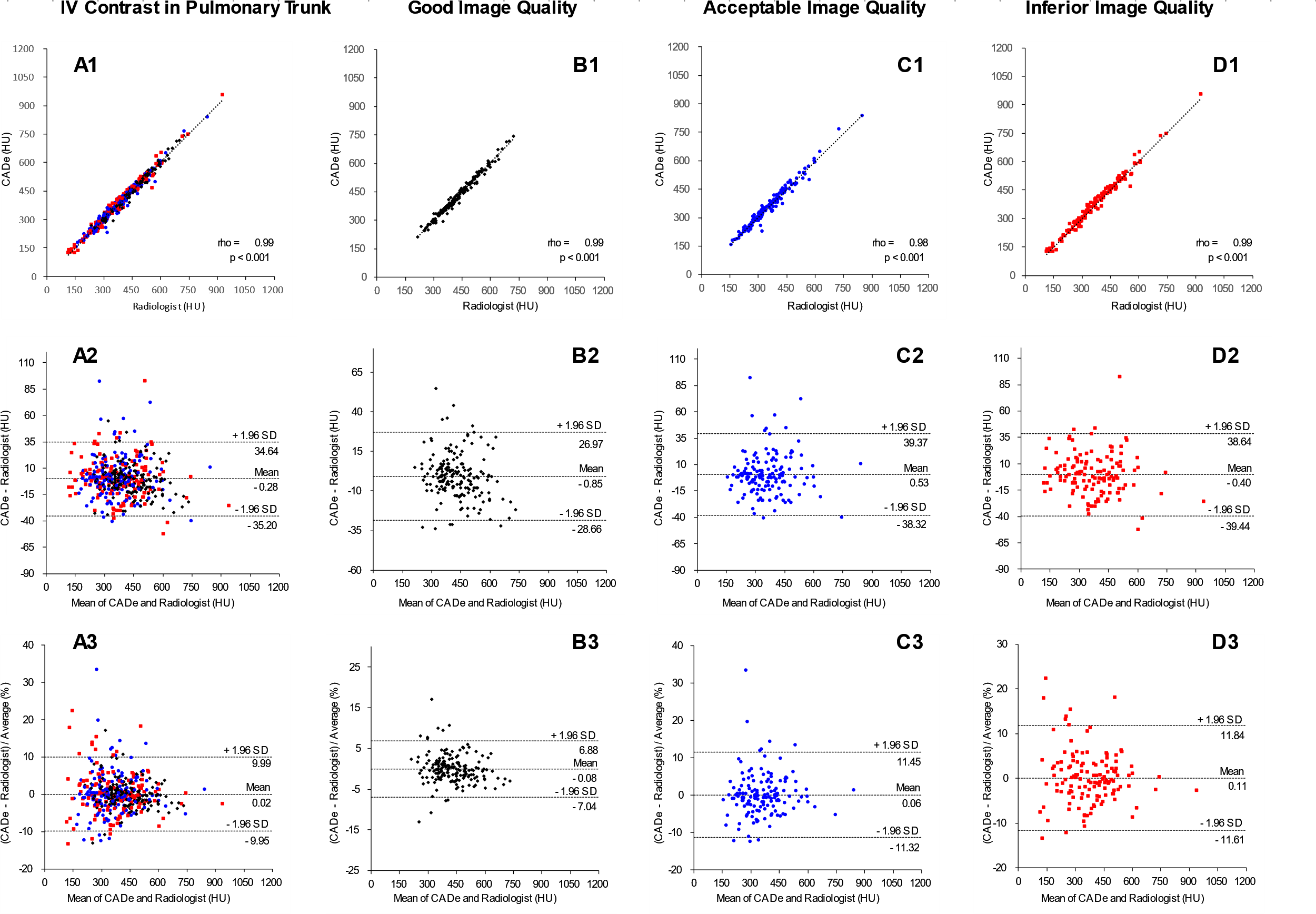


**Supplemental Figure 18.** **High performance in intravenous contrast agent measurement in the pulmonary trunk independent of image quality.** Automatic and manual measurements of test set cases were compared by regression analysis (top row, dashed regression lines), and Bland-Altman plots of differences in Hounsfield units (middle row, limits of agreement from -1.96 to +1.96 SD) and Bland-Altman plots of differences in percentage (bottom row, limits of agreement from -1.96 to +1.96 SD). A. Images of all qualities (n=455 CTPA exams). B. Good image quality (n=179 CTPA exams). C. Acceptable image quality (n=146 CTPA exams). D. Inferior image quality (n=130 CTPA exams). The quality of the CTPA examinations was assessed by the radiologist as good (black diamonds), acceptable (blue circles), or inferior (red squares).


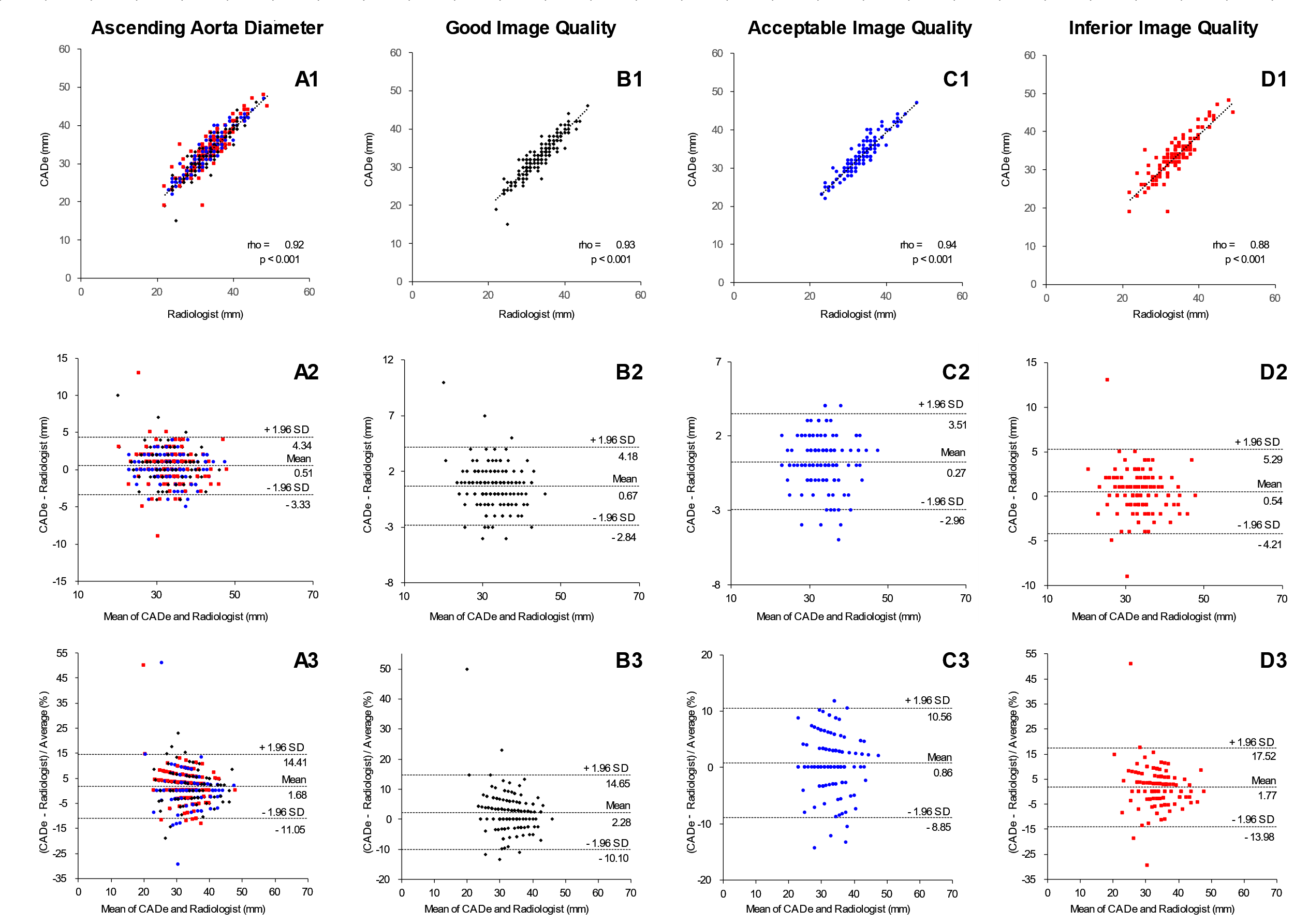


**Supplemental Figure 19.** **Image quality dependent performance in ascending aorta diameter measurements.** Automatic and manual measurements from test set cases were compared by regression analysis (top row, dashed regression lines), and Bland-Altman plots of differences in diameters (middle row, limits of agreement from -1.96 to +1.96 SD) and Bland-Altman plots of differences in percentage (bottom row, limits of agreement from -1.96 to +1.96 SD). A. Images of all qualities (n=447 CTPA exams). B. Good image quality (n=176 CTPA exams). C. Acceptable image quality (n=143 CTPA exams). D. Inferior image quality (n=128 CTPA exams). The quality of the CTPA examinations was assessed by the radiologist as good (black diamonds), acceptable (blue circles), or inferior (red squares).


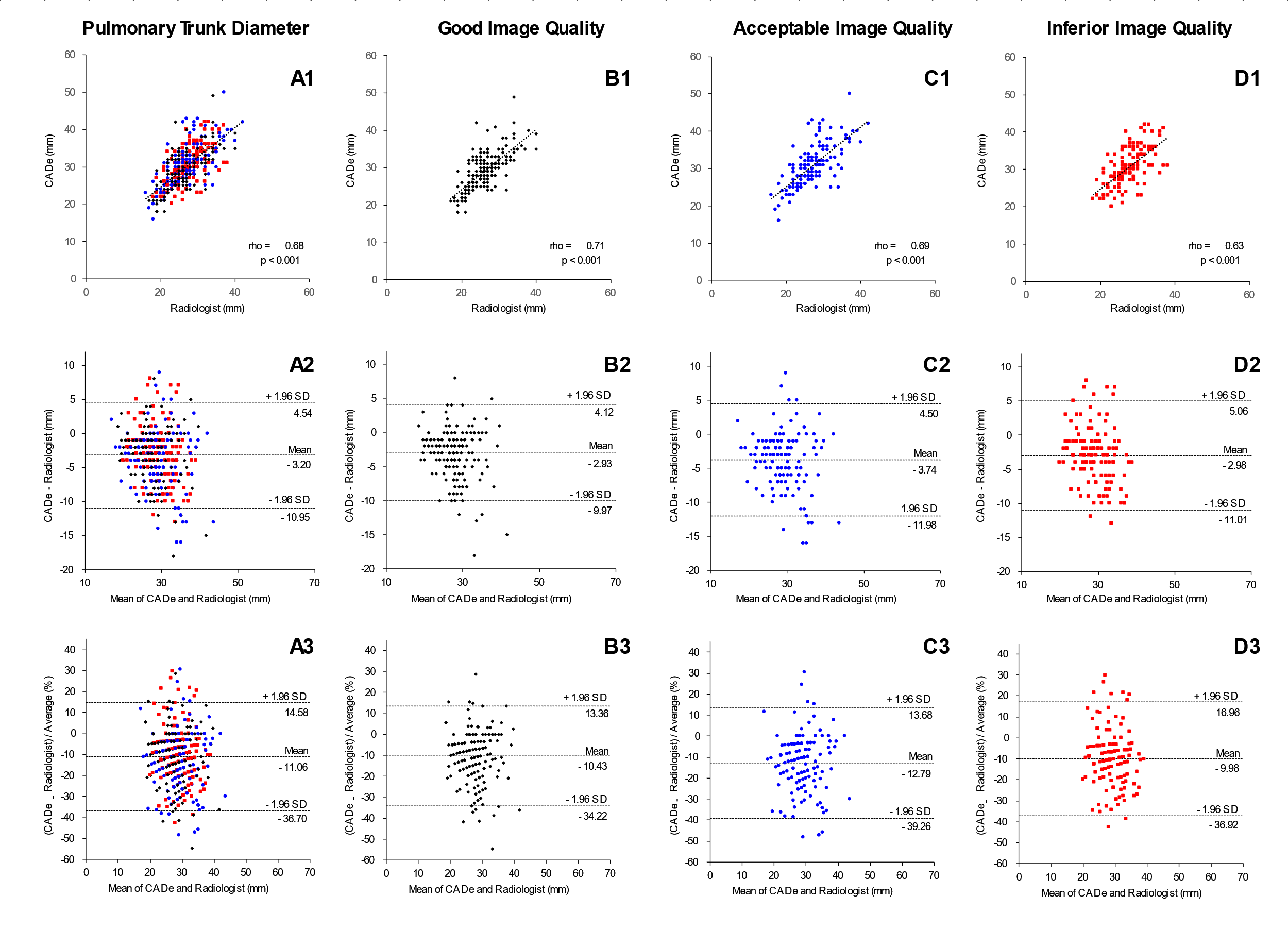


**Supplemental Figure 20. Image quality dependent performance in pulmonary trunk diameter measurements.** Automatic and manual measurements from test set cases were compared by regression analysis (top row, dashed regression lines), and Bland-Altman plots of differences in diameters (middle row, limits of agreement from -1.96 to +1.96 SD) and Bland-Altman plots of differences in percentage (bottom row, limits of agreement from -1.96 to +1.96 SD). A. Images of all qualities (n=455 CTPA exams). B. Good image quality (n=179 CTPA exams). C. Acceptable image quality (n=146 CTPA exams). D. Inferior image quality (n=130 CTPA exams). The quality of the CTPA examinations was assessed by the radiologist as good (black diamonds), acceptable (blue circles), or inferior (red squares)

**
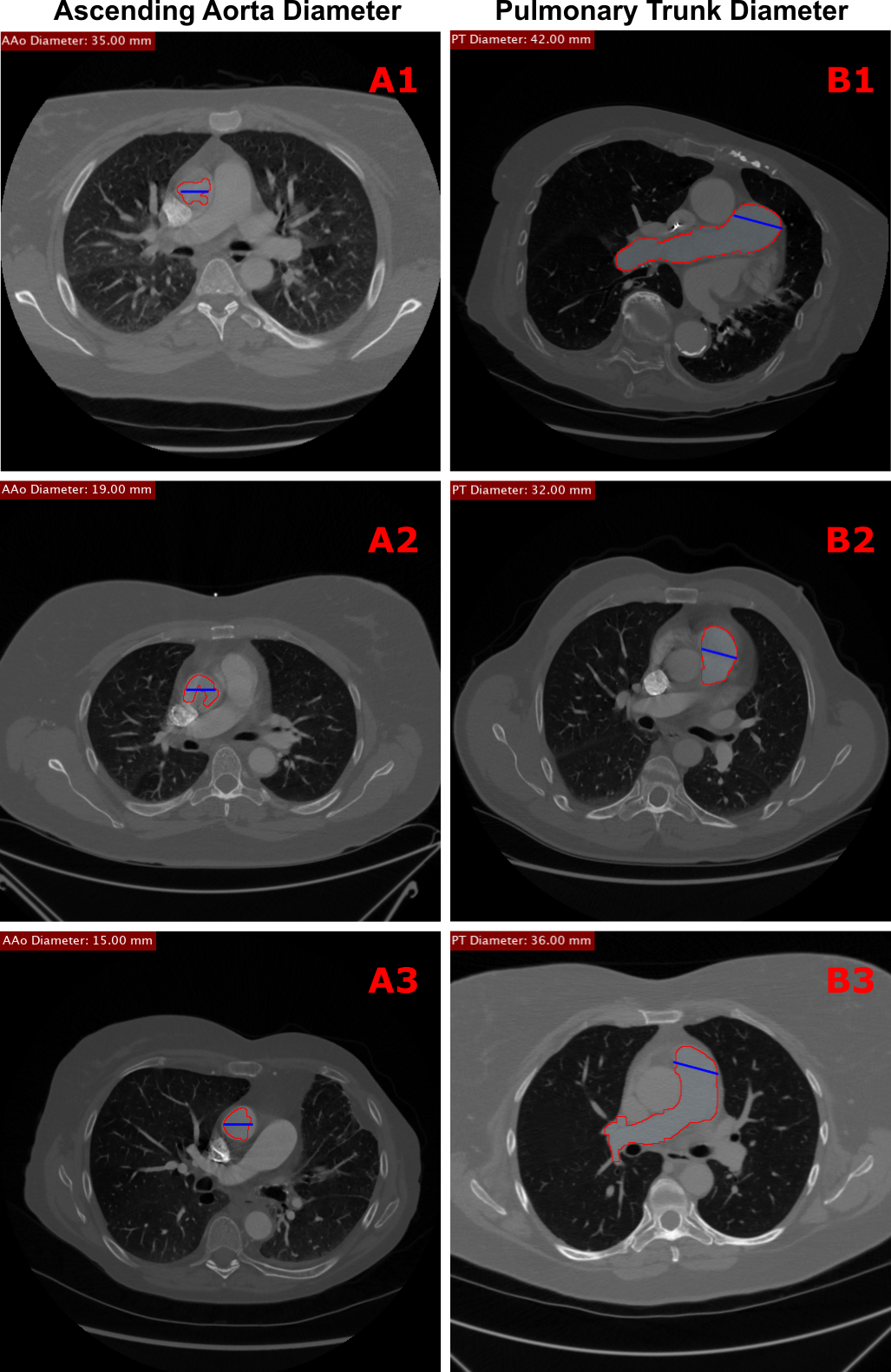
**

**Supplemental Figure 21. Examples of incorrect CADe measurements on correctly detected mediastinal structures.** A. Ascending aorta diameter (A1: CADe measurement 35 mm, radiologists 26 mm; A2: CADe 19 mm, radiologists 32 mm; A3: CADe 15 mm, radiologists 25 mm). B. Pulmonary trunk diameter (B1: CADe 42 mm, radiologists 32 mm; B2: CADe 32 mm, radiologists 22 mm; B3: CADe 36 mm, radiologists 26 mm). Red line, CADe detection results; blue line, CADe diameter measurement.


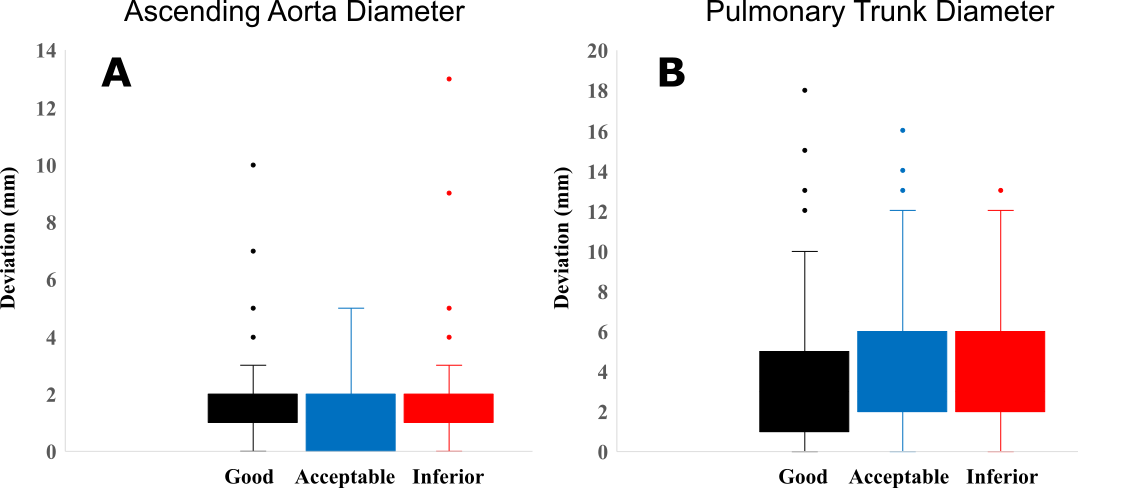


**Supplemental Figure 22.** **Image quality dependent diameter measurement deviation between the CADe system and the radiologist.** A. AAo diameter (mean deviation for good examinations 1.39 mm, acceptable 1.22 mm, inferior 1.77 mm). B. Pulmonary trunk diameter (mean deviation for good examinations 3.44 mm, acceptable 4.32 mm, inferior 4.15 mm).

***
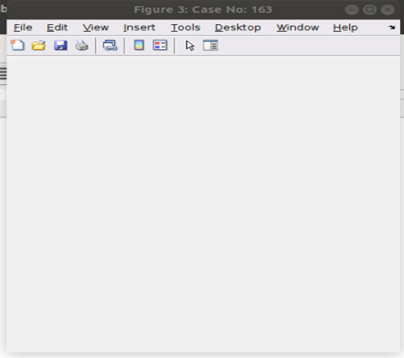
***

**Supplemental Figure 23. An example of a blank image.** This case is labeled as failed.


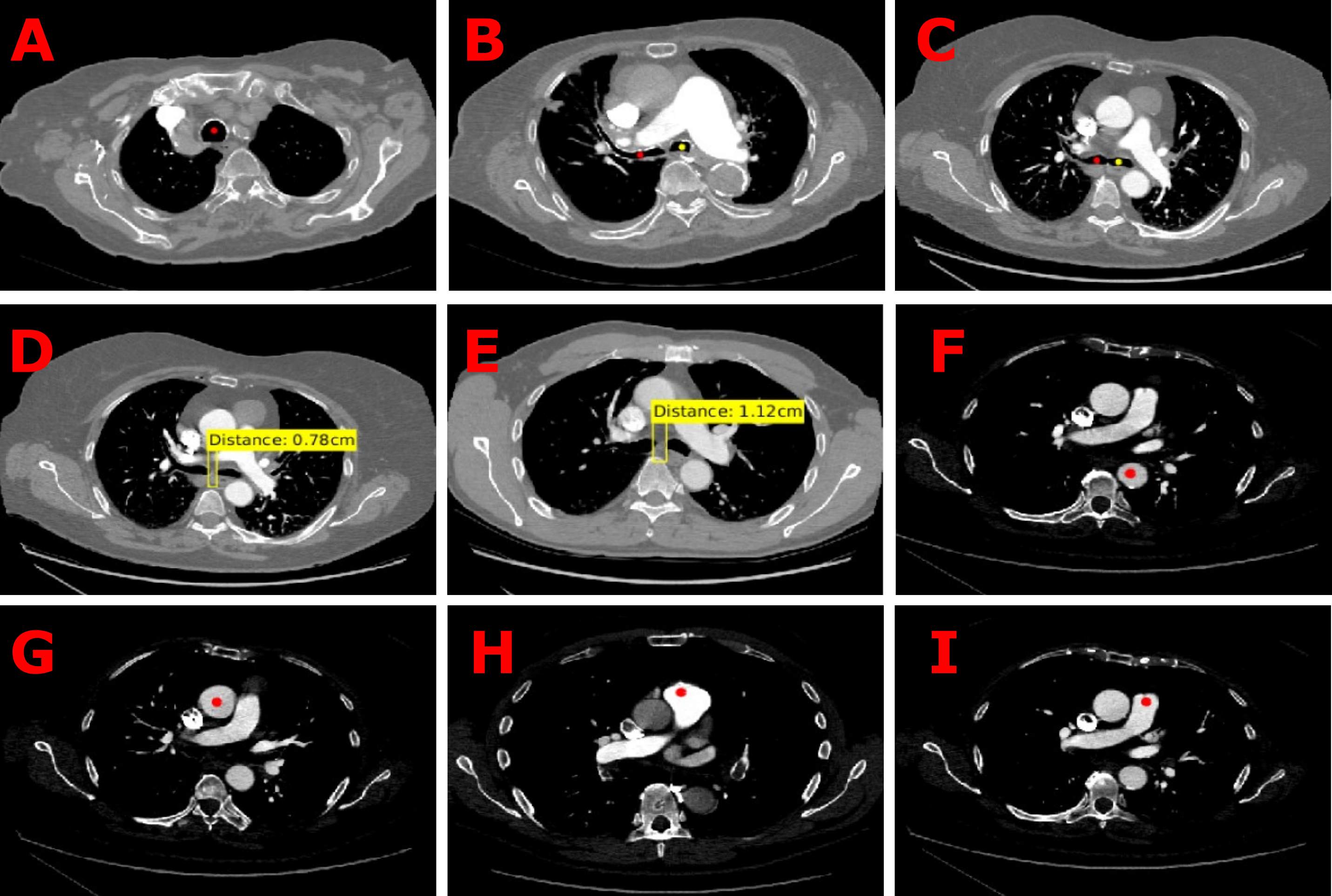


**Supplemental Figure 24. Examples of successful detection of compartments.** A. Trachea. B-C. The left and the right main bronchus. D-E. The carina level. The distance between the left main bronchus and the right main bronchus is in the predefined interval (0.7 cm - 1.7 cm). F. Descending aorta. G. Ascending aorta. H. The proximal part of the pulmonary trunk/pulmonary valve. I. Pulmonary trunk. The markers (red or yellow) are inside desired organs.


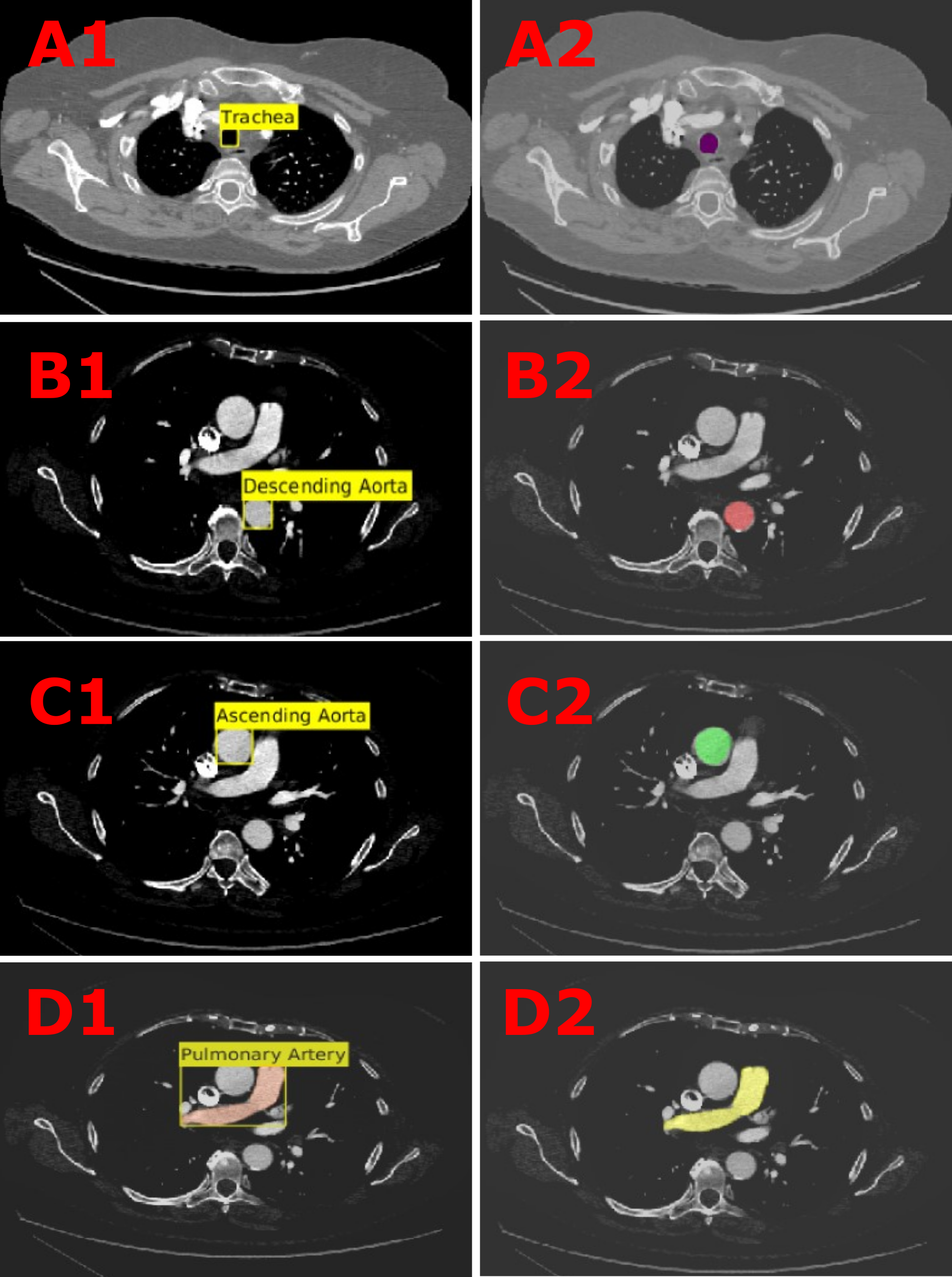


**Figure 25. Examples of successful segmentation of compartments.** A. Trachea B. Descending aorta. C. Ascending aorta. D. Pulmonary trunk/pulmonary valve. In the left column, the segmentation is shown by a bounding box. In the right column, the segmentation is illustrated by a mask.

**SUPPLEMENTAL METHODS**

**Image quality score calculation**

Image quality (Q) in CTPA exams is affected by several aspects such as motion-breathing artifacts, streak artifacts, image noise, contrast concentration, and lung parenchymal diseases. If each aspect can be scored on a point scale and if each aspect has a different weighting factor (*k*) on image quality then the image quality can be calculated as the sum of these scores (s) and formulated as follows:

| $Q(s)= \sum_{i=1}^{N} {k_{i}*ps}_{i}$ | (D.1) |
| --- | --- |

where *ps* represents the point scale of the aspect and $N$ is the total number of aspects.

The aspects, point scales and weighting factors were determined subjectively by radiologist TF. Selected aspects with point scales and weighting factors are given in Supplemental Table 1. Using equation (D.1) with the parameters of Supplemental Table 1, the image quality score *s* can be determined as:

$s= k_{1}*mba+ k_{2}*sa+k_{3}*n+k_{4}*d+ k_{5}*ld$ (D.2)

where $k_{1}, \ldots, k_{5}$ are weighting factors and $mba$,$sa$, $n$, $d$, and $ld$ represent the aspects motion-breathing artifacts, streak artifacts, image noise, contrast concentration, and lung disease, respectively.

Each CTPA examination was assigned to one of three classes of quality (good, acceptable, inferior) based on the total score in equation (D.2).

Finally, the quality of exam (Q) can be formulated as follows:

| $Q=\left\{ \begin{aligned} good, &if 0\leq s \leq3 \\ acceptable, &if 4\leq s \leq7 \\ inferior, &if 8\leq s \end{aligned} \right.$ | (D.3) |
| --- | --- |

**Description of the CADe system**

**Note:** Unless otherwise stated, all image processing and analysis techniques were performed on the axial (transverse) plane.

1. **Calculating linear scale value / Converting CT Data to Hounsfield Units**

In a first step, every voxel in the CT Data (original linear attenuation coefficient) was converted to the Hounsfield Units scale by the formula HU = pixel_value * slope + intercept where the slope and intercept values are found in the DICOM header file tag (0028, 1053) and (0028, 1052), respectively.

1. **Finding the scanning direction**

Depending on the CT examination procedure, the patient can be scanned in cranial to caudal (head to tail or superior to inferior) or caudal to cranial (tail to head or inferior to superior) direction. The scanning direction can be calculated by comparing the first and last CT slice image position information in the DICOM header file tag (0020, 0032). The smaller z-axis value of the first and last slice indicates the caudal part of the patient.

1. **Calculating the orientation of the CT scan**

The system first locates the 3D positions of two anatomical landmarks, carina of trachea and an apical level of the pulmonary valve, to find seed points of mediastinum structures (ascending and descending aorta, pulmonary trunk). These anatomical landmarks are detected automatically by the system scanning CTPA volume images in the cranial to caudal direction. Hence, knowing the scanning direction is crucial to detect the anatomical landmarks.

Further, the orientation of the patient in the CTPA image with respect to the x-axis of the image plane is a key factor to automatically and accurately detect the carina of trachea, the apical level of the pulmonary valve, the ascending and descending aorta and the pulmonary trunk. The orientation of the patient is the angle between the major axis of the patient’s image and the x-axis of the image plane (Supplemental Figure 2). To achieve accurate detection, the major axis of the patient in the image must be parallel to the x-axis of the image plane. The first image in a CT volume may contain information about the volume rather than an actual image of the patient (i.e., CT Topogram). Therefore, the cranial orientation of the patient is first computed using the second image of the CT volume images (Supplemental Figure 3).

1. **C****hecking cranial to caudal continuity of patient orientation**

Patient orientation may vary along the superior to inferior direction in the CTPA study because of scoliosis, patient movement during the examination, the patient lying down obliquely on the CT table during the examination, or other reasons. Curvature on just a few slices on the caudal or cranial part of the CTPA examination does not interfere with the proper functioning of the developed algorithm. However, if 𝜶≥𝟏𝟖 (Supplemental Figure 3) in any of the first slices (top 10 slices) in the CTPA study, such as the second slice, an additional orientation test is performed on the caudal part of the patient. When computing patient orientation with respect to the x-axis (step 3), the anatomy of the patient may preclude correct orientation (Supplemental Figure 4).

Therefore, we developed a new method to calculate patient orientation more precisely based on the caudal slices of the CT exam, as their orientation was found to correlate closer to the orientation of the carina of trachea and pulmonary valve (Supplemental Figure 5). The slope of the curves in region 3 or 4 (Supplemental Figure 5, steps 4.6 and 4.7) is almost parallel to the orientation of the spine (Supplemental Figure 6). Further, the orientation of the spine is almost the same as the orientation of the patient, even for patients with scoliosis. Therefore, one way to find the orientation of the patient is to calculate the orientation of the spine. To do this, the spine must first be detected in the CT volume, and the entire spine must be segmented correctly. However, calculating the orientation of the patient in this way is computationally challenging and hard to validate. With the method proposed in Step 3 and 4, we can easily calculate the orientation. In summary, to decide whether to apply rotation to the image or not, we first check the angle α in the cranial part of the patient by the method of Step 3. If the cranial α > 17°, we compute the angle α in the caudal parts of the patient by the method of Step 4. Finally, all CT scans of the CTPA exam are rotated according to the angle α computed in Step 4.

1. **Detecting the trachea in a CT volume**

In a majority of CT exams, the trachea can be located as a tubular air-filled structure of lower HU density than surrounding lung tissue. However, in a minority of CT exams, there are artifacts in the CT planes such as beam hardening, craniofacial structures or upper airway structures that resemble the trachea. Finding the trachea in presence of such artifacts has received little attention in the literature. Here, we have developed a fully automated algorithm capable of detection of trachea with or without presence of artifacts.

- 1. To accelerate computation, the CT volume $I(x,y,z)$ is downscaled to half its size with respect to the x- and y-axes but not the z-axis $I(\frac{x}{2},\frac{y}{2},z)$. In some CT exams, the trachea adjoins the right lung before the carina of the trachea because of the curvature of the trachea. This prevents localization and segmentation of the trachea. By empirical observation, such adjoining did not occur in the cranial 15% of CT slices in the training set. To correctly localize the trachea in 3D, the system therefore generates two consecutive volumes of interest where trachea candidates are generated and assessed.
  2. First, the cranial of the two consecutive volumes of interest, volume of interest 1 (VOI1) is determined by extracting the CT slices starting from the second slice ($s_{2}$) of the CT exam up to $s_{n}$ in the cranial to caudal direction, where $s_{n}$ = 15% of the total number of slices in the CT scan.
  3. Air areas (lungs, airways, and artefacts that resemble air) are segmented in VOI1 by applying the following steps to each slice of VOI1:
     1. The slice is thresholded over -300 HU.
     2. 2D connected component analysis is applied.
     3. The component with the largest area is designated as the thoracic cavity.
     4. A morphological flood-fill operation is applied.
     5. To get air filled areas, a logical AND operation is applied to the output of step 5.1. and the output of step 5.3.4.
  4. Next, a pool of potential trachea candidates (TCP1) in VOI1 is generated. The average HU density of VOI1 is calculated. All slices in VOI1 are then thresholded with this average density. Empirically, the area of the trachea was found to be within the range 10-1200 pixels in the CT slices of the training set. Therefore, components with an area < 10 or > 1200 pixels are excluded in each slice. To remove false positive trachea candidates, volumes < V voxels are excluded where V is calculated by:

$$V=\left( \left| s_{n}- s_{2} \right|+1 \right)*7$$

where $s_{2}$ and $s_{n}$ are calculated in above is an empirically determined coefficient to calculate likely minimum volume of trachea candidates.

When the steps 5.1 to 5.4 are applied to the CT volume, most often more than three 3D components (trachea, esophagus, right lung, and left lung) are obtained, but in some cases only two 3D components (trachea, right or left lung) are obtained. If there is only one 3D component in the TCP1 as a result of above steps, this component is designated as the trachea. However, if there is more than one component in the TCP1, we do not know which component is the trachea, the right lung, the left lung or the esophagus. Therefore, to recognize the trachea correctly and automatically the following steps are applied:

- 1. A second volume of interest (VOI2) to search for the trachea is defined.

The volume starting from the last slice of VOI1 ${(s}_{n})$ up to slice $s_{k}$ is calculated by

$$s_{k}=m-m*0.25$$

where *m* is half of the total number of slices in the CT volume and the empirically determined coefficient 0.25 is used to calculate the total number of slices to be included in VOI2 based on empirical observation from the training set. The VOI2 will contain the right and the left lung, except when one of the lungs is completely collapsed.

- 1. Air areas (lungs, airways, and artefacts that look like air) are segmented in VOI2 by applying the following steps to each slice of VOI2:
     1. The slice is thresholded over -300 HU.
     2. 2D connected component analysis is applied.
     3. The component with the largest area is designated as the thoracic cavity.
     4. A morphological flood-fill operation is applied.
     5. A logical AND operation is applied the output of step 5.6.1 and the output of step 5.6.4 in order to get the air filled areas.
  2. A pool of potential trachea candidates (TCP2: trachea candidates pool 2) is generated for VOI2
     1. The average HU density of VOI2 is calculated.
     2. The average HU density computed in 5.7.1 is multiplied by 0.05 which is a HU density enhancement factor determined by empirical observation.
     3. All slices in VOI2 are thresholded with the HU density computed in 5.7.2.
     4. Areas < 10 and > 1200 pixels in each slice are excluded.
     5. Volumes < V voxels are excluded and V is calculated by:

$$V=\left( \left| s_{k}- s_{n} \right|+1 \right)*7$$

where $s_{k}$ is calculated in step 5.5 and $s_{n}$ is calculated in step 5.2 and 5.7 is an empirically determined coefficient to calculate likely minimum volume of trachea candidates.

- 1. Choosing the right component that is the trachea.

To mark some 3D components to potentially be the trachea (TCP1) we limited the search space (VOI1) in steps 5.1 to 5.4. However, we do not know which component in the TCP1 is the trachea. Therefore, in steps 5.5 to 5.7 we generate a second search space (VOI2) from the CT scan, which is consecutive of VOI1 in cranial to caudal direction, to limit the trachea candidate pool. Anatomically in VOI1+VOI2, the longest 3D component in cranial to caudal direction is the trachea. In the following steps, we combine TCP1 and TCP2 to detect the longest component.

- - 1. Each 3D connected component in the TCP1 is labeled using the bwlabeln function in Matlab R2019b.
    2. Each 3D connected component in the TCP2 is labeled using the bwlabeln function in Matlab R2019b.
    3. To find common components between TCP1 and TCP2, a logical AND operations is applied to the outputs of step 5.8.1 and step 5.8.2 at slice $s_{n}$, which is the only common slice between the two volumes.
    4. To obtain all components related to TCP2, a logical AND operation is applied the output of step 5.8.1 and step 5.8.3.
    5. To obtain all components related to TCP1, a logical AND operation is applied the output of step 5.8.2 and step 5.8.3.
    6. The outputs of step 5.8.4 and 5.8.5 are combined by arithmetic addition.
    7. The longest component, defined as the component spanning the largest total number of CT slices in the combined volume of 5.8.6, is designated as the trachea.
    8. For further steps, we must choose one of the CT slices along the z axis where the trachea is located by:

$$s_{t}=round\left( \frac{\min\left( p \right)+\max\left( p \right)}{2} \right)$$

where $p$ is a matrix containing positions of the trachea along the z-axis.

1. **Detecting tracheal intubation**

Some patients undergo CT examination with tracheal intubation. The insertion of an endotracheal tube creates changes in appearance of the trachea in CT examinations and interferes with automatic detection of the carina of trachea. To overcome this problem, tracheal intubation needs to be detected by the system. If the trachea was successfully located in step 5, tracheal intubation is detected by the following steps;

- 1. The center point of the trachea is calculated by the bounding box or minimum bounding rectangle method. As the trachea appears as a circular object in a transverse CT slice, the center point of the smallest rectangle containing the trachea is the same as the center point of the trachea.
  2. Since the HU density of the inserted tube is high, the CT image at slice $s_{t}$ from step 5.8.8 is thresholded over 300 HU.
  3. Areas < 15 and > 100 pixels in the binary image of step 6.2 are excluded.
  4. 2D connected component analysis is applied to the binary image of step 6.3.
  5. The center points of all distinct 2D connected components in the binary image are calculated. If a tracheal intubation exists, then the endotracheal tube is represented by one of these 2D connected components.
  6. The endotracheal tube is located inside of the trachea. Therefore, the center points of the tube and the trachea must be close to each other. To find the closest components to the trachea, the Euclidean distance of component center points to the center point of the trachea is calculated.

$$d\left( x,y \right)= \sqrt{\sum_{i=1}^{n} {(x_{i}-y_{i})}^{2}}$$

where $n$ is the total number of components and $x$ and $y$ are the center points of the trachea and the components respectively.

- 1. If the distance of the center points of 2D connected components to the center point of the trachea is < 11 pixels then these components are marked as potential endotracheal tubes.

In some cases, calcification can occur around the trachea and the HU density and morphology of the calcification may appear similar to an endotracheal tube. Therefore, additional filters are required to ensure that the detected component is an endotracheal tube. The average HU density of the 2D component is calculated, and if > 900 HU the component is designated as an endotracheal tube. While the HU density of a calcification may be > 900 HU in theory, we have never noticed any calcification around the trachea where the HU density of the calcification is > 900 HU in areas > 15 pixels in the training set. Once an endotracheal tube is detected inside the trachea, we need to find the CT slice where the intubation does not exist in order to track tracheal areas up to the carina of trachea accurately. The location of the new CT slice must be between the end of tracheal intubation and the carina of the trachea. We find this location by the following steps;

- 1. The 3D region growing method is applied to the CT scan in order to extract the tracheal intubation. The seed points for the 3D region growing method are generated from step 5.
  2. The last CT slice number of the tracheal intubation in the cranial to caudal direction is obtained. This location is set as the preferred CT slice number ($s_{l}$) for tracking the trachea up to the carina trachea.

1. **Segmentation of airways**

Since we have located the trachea before the carina in cranial to caudal direction in the CT stack $I\left( x_{t},y_{t},z_{t} \right)$, we can now track the trachea up to the bifurcation point by comparing trachea regions slice by slice. To continue the tracking process, we need to segment the trachea region. We can segment the first trachea region by the 2D region growing method. The inputs of the region growing method are the binary image and seed points. First, the CT slice $I\left( :,:,z_{t} \right)$ is thresholded over -700 HU to acquire a binary image (step 7.1). With this thresholding process we only focus on airway voxels. While a threshold of -300 HU can be used instead of -700 HU, a threshold of -700 prevents the lung and airway areas from joining each other. Second, the location of the trachea in the 3D stack $I\left( x_{t},y_{t},z_{t} \right)$ obtained from step 5 (if intubation has been detected, the location of the trachea was obtained from step 6) will be designated as seed points (step 7.2). We then apply 2D region growing using these seed points ($x_{t} and y_{t}$) to segment the trachea region.

Prominent morphological changes in the trachea regions between two consecutive CT slices indicates a bifurcation point. Thus, once we have segmented the trachea, we move to the next CT slice $I\left( :,:,z_{t+1} \right)$ in cranial to caudal direction to examine for signs of a bifurcation point We threshold the new CT slice over -700 HU (step 7.3) and a logical AND operation is applied to this new binary image with the segmented trachea region (step 7.4). The 2D region growing method is applied to the output of step 7.4 (step 7.5). The area of the segmented trachea is calculated as the total number of pixels in the segmented 2D component (step 7.6). Empirically, the area of the airways in 2D was smaller than 1750 pixels before the bifurcation of the trachea in the training set. However, if the calculated area exceeds 1750 pixels, the trachea is adjacent to the lungs and then we apply watershed transform to segment the trachea from the lungs (step 7.7). If the calculated area is smaller than 1750 pixels or once watershed transform is applied to the image, the trachea region in the previous CT slice $I\left( :,:,z_{t} \right)$ is subtracted from the newly segmented trachea region in the current CT slice $I\left( :,:,z_{t+1} \right)$ (step 7.8).

The difference between the newly segmented trachea region and the trachea region in the previous CT slice is the presence of the one of the main bronchi. We look for two prominent morphological changes to ensure that the component is one of the main bronchi. The first morphological change investigated is the mean densities of the components (components 1 and 2 represent the segmented trachea region in the current CT slice and the difference of the two images, respectively) and the other morphological change investigated is the size of the components. First, the mean densities of the components are calculated (step 7.9). If the absolute value difference of the average density values ​​of the components is less than 400 HU, we are looking for the below condition in order to be sure that the difference between two images represents the presence of one of the main bronchi:

$$f\left( x \right)=\left\{ \begin{aligned} 1, A_{c}>( \frac{A_{t}}{k} )& \\ 0, otherwise & \end{aligned} \right.$$

where, $A_{c}, A_{t}$ is the area of component 2 and the area of the trachea region in the previous CT slice respectively. The k is the divide factor which is calculated as:

$$k=\left\{ \begin{aligned} 5, A_{t}>400 )& \\ 4, otherwise & \end{aligned} \right.$$

The cut-off value 400 was determined by empirical observation on the training set.

Once the main bifurcation of trachea is detected, two components are obtained. One is the right main bronchus and the other is the left main bronchus. We then calculate the center points of these components and designate the left and the right main bronchus by comparing y-axes of the mass center of the components.

1. **Finding the optimal carina location**

Since the carina trachea is a 3D structure, we need to designate a slice which represents the carina trachea in 2D. We designated the CT slice where the distance between the left main bronchus and the right main bronchus is at least 0.75 cm as the optimal carina location $I\left( :,:,z_{c} \right),$ with the distance calculated by taking differences of vertical coordinates (y-axes) of the left most pixel of the right main bronchus ($P_{r}$) and the right most pixel of the left main bronchus ($P_{y}$). This difference calculation operation is done in each slice starting from the where bifurcation occurred until the ${|P}_{r}- P_{y}|$ is equal or greater than 0.75 cm.

1. **Detecting the descending aorta**

The descending aorta is located posterior to the left main bronchus. Since we detected the left main bronchus in Step 7, we start from a line drawn from the center of the left main bronchus and that cuts the x-axis at an angle of 22.5 degrees downwards clockwise. Thereafter, we draw 8 lines, each 100 pixels long, with an angle of 7.5 degrees between each other (Supplemental Figure 8) to find the descending aorta (SS1: search space 1). However, in some cases, the descending aorta is shifted toward the right side. Therefore, we need to shift the search space to the right. We create a second search space (SS2: search space 2) by starting from an artificial line that cuts the x-axis at an angle of 60 degrees downwards clockwise from the center of the left main bronchus, and drawing 9 lines, 100 pixels long, with an angle of 7.5 degrees between each other (Supplemental Figure 8). Those generated rays are used for obtaining pixels from tissues they pass over.

Once we have generated artificial rays, we apply the following steps ray by ray in order to locate the descending aorta (Supplemental Figure 7). First, we collect all pixels on the ray. Second, we threshold those pixels over –1 HU to acquire a binary image. Third, we apply 2D connected component analysis to find the largest connected pixels on the ray. Fourth, we calculate the total number of pixels on the segmented component. If the total number of pixels is greater than 15, then we calculate mean x and y coordinates of those pixels, separately, in order to reduce to one data point. If the above criterion is met only by one ray, in other word, if we only have one x and y coordinate ($P_{desAo}$), we assume that the pixel in this coordinate belongs to the descending aorta. Otherwise, if the above criterion is met by more than one ray then we applied k-means clustering algorithms to the only x coordinates (since the rays radiate on x-axis) in order to cluster rays into two groups. After clustering the x coordinates, we compared the number of elements between the clusters. The cluster with the most elements is designated as cluster 1 and the other one is designated as cluster 2. Then the x coordinates in each cluster and the y coordinates of these x coordinates are averaged to reduce one data point ($P_{desAo1}, P_{desAo2})$.

Once we designate a reference point ($P_{desAo1}, P_{desAo2})$ which possibly belongs to the descending aorta, we apply the following steps to segment and track the descending aorta slice by slice.

To separate tissues from each other and to increase segmentation accuracy we obtain three binary images. The first image is used to identify high density tissues ($I_{1}$), the second is used to determine the edge and borders of the tissues ($I_{2}$, Supplemental Figure 9), and the third binary mask is used to determine the voxel of interest ($I_{3}$).

To identify high density tissues, we threshold the image over 750 HU density ($I_{1}$). To determine the edge and borders of the tissues on the image, we apply the following seven sub steps. We first apply Gaussian filtering to remove noise and smoothen the image ($I_{\sigma}$, step 9.1). Second, we calculate second-order partial derivatives of the image and organize them into a Hessian matrix ($H$) as formulated below (step 9.2);

$$H_{ij}=\frac{\partial^{2}f}{\partial x_{i}\partial x_{j}}= \left( \begin{matrix} \frac{\partial^{2}f}{\partial x_{1}^{2}} & \cdots& \frac{\partial^{2}f}{\partial x_{1}\partial x_{n}} \\ \vdots& \ddots& \vdots\\ \frac{\partial^{2}f}{\partial x_{n}\partial x_{1}} & \cdots& \frac{\partial^{2}f}{\partial x_{n}^{2}} \end{matrix} \right)$$

Third, we calculate the Eigen value ($\lambda_{1}$) of the Hessian matrix (step 9.3). Fourth, we calculate the average value ($\mu_{\lambda}$) of $\lambda_{1}$ and then threshold $\lambda_{1}$ over $\mu_{\lambda}$ to obtain a binary image (step 9.4). Fifth, we apply Canny edge detection to the smoothed image ($I_{\sigma}$) (step 9.5), followed by morphological dilation (step 9.6). Finally, we combine the results of step 9.4 and step 9.6 into one single image ($I_{2}$).

To determine the voxel of interest (all potential voxel candidates of the descending aorta in the smoothed image), we first calculate the mean HU density ($\mu$) of the smoothed image ($I_{\sigma}$). According to the value of the calculated mean density, we enhanced the mean density ($\mu_{e}$) by

$$\mu_{e}=\left\{ \begin{aligned} \mu-60, \mu>160 HU \\ \mu-30, \mu>130 HU \\ \mu-15, \mu>100 HU \\ \mu, otherwise \end{aligned} \right.$$

The numbers in the above formula were determined by empirical observation on the training set. We then thresholded the smoothened image ($I_{\sigma}$) with the calculated mean density ($\mu_{e}$) and obtained a binary image ($I_{3}$).

To search for the descending aorta, we designate a region of interest (Supplemental Figure 10) by the formula:

$$I_{P}=\left( I_{3}- I_{2}- I_{1} \right)>0$$

Once we have determined the region of interest for the descending aorta searching, we apply 2D connected component analysis. Empirically, the area of the descending aorta was found to be above 200 pixels in the training set. Therefore, components with an area < 200 pixels were excluded. Further, we calculate the mass center of the components to measure the Euclidean distances between the components and the reference point $P_{desAo1}$. If the measured minimum Euclidean distance is > 12.5 for the reference point $P_{desAo1}$, then we measure the Euclidean distances between the components and the reference point $P_{desAo2}$. Next, we designate the closest component to the reference points ($P_{desAo1} or P_{desAo2})$ as a potential descending aorta. We use the mass center of the closest components to the reference points as a seed point ($P_{desAoSeed}$). To detect descending aorta from potential candidates we check circularity (or sphericity) of the components by the formula below,

$$circularity =\left( 1-ecc \right)* \frac{4\pi A}{P^{2}}$$

where $A$ and $P$ are the area and the perimeter of the component respectively, and the eccentricity ($ecc$) is a constant expressed as the ratio of the distance between the foci of the object and its major axis length. The output of the formula is between 0 and 1. A component whose $circularity$ is 1 is a circular object. We then assign the component with the biggest circularity as the descending aorta. Once we obtain the descending aorta, we draw a 1 ${cm}^{2}$ circle, the center point of which is the same as the mass center of the descending aorta. We then calculate the average HU density of this circle.

1. **Detecting the aortic arch**

In step 9, we located the descending aorta at the carina level $I\left( :,:,z_{c} \right)$. We can then track the descending aorta up to the aortic arch $I\left( :,:,z_{aa} \right)$ in the caudal to cranial direction. Prominent morphological changes in descending aorta regions between two consecutive CT slices indicates that we have reached the aortic arch. Therefore, we need to segment all descending aorta regions $I\left( :,:,z_{c}:z_{aa} \right)$ between the carina of the trachea and the first prominent appearance of the aortic arch in the CT stack. We segment and compare the descending aorta regions slice by slice by using the following methods:

- 1. Gray scale segmentation
  2. Anisotropic diffusion filtering, by
     $I_{t}=div\left( c\left( x,y,t \right)\nabla I \right)=c\left( x,y,t \right)\Delta I+ \nabla c\cdot\nabla I$
  3. Eigen values of Hessian matrix
  4. Canny edge detection
  5. Morphological operations
  6. 2D region growing

1. **Detecting the ascending aorta**

Once we have located the aortic arch, we can track the anterior part of the aortic arch (Supplemental Figure 11) in the cranial to caudal direction in order to reach the ascending aorta. Anatomically, tracking the anterior part of the aortic arch for a few CT slices (between 2 to 7 slices) is sufficient for reaching the ascending aorta. Thus, we track the anterior part of the aortic arch for 3 slices starting from the first descending aorta regions in the CT stack in cranial to caudal direction. The anterior part of the aortic arch is segmented by taking the difference of the first appearance of aortic arch in the CT stack from the first descending aorta regions in the CT stack which are extracted in the previous steps. We then track the anterior regions of the aortic arch slice by slice in cranial to caudal direction by extracting regions using the following steps:

- 1. Gray scale segmentation
  2. Anisotropic diffusion filtering
  3. Eigen values of Hessian matrix
  4. Canny edge detection
  5. Morphological operations
  6. 2D region growing

1. **Extracting the ascending aorta**

In step 11, we located the ascending aorta in the CT stack $I\left( :,:,z_{aa+2} \right)$. Our aim here is to segment all the ascending aorta regions between the first located slice $I\left( :,:,z_{aa+2} \right)$ and the carina trachea $I\left( :,:,z_{c} \right)$. Within this volume of interest $I\left( :,:,{z_{c}:z}_{aa+2} \right)$, we segment and track the ascending aorta slice by slice using the following methods:

- 1. Gray scale segmentation
  2. Anisotropic diffusion filtering
  3. Eigen values of hessian matrix
  4. Canny edge detection
  5. Morphological operations
  6. 2D region growing

In every slice, we measure the diameter of the segmented ascending aorta. Finally, we calculate the diameter of the ascending aorta as the mean of the diameters from each slice.

1. **Detecting the pulmonary trunk**

Anatomically, the pulmonary trunk appears next to the ascending aorta in the CT slice. Once we have located the ascending aorta, we can therefore locate the pulmonary trunk by creating a rectangular search space adjacent to the ascending aorta based on the ascending aorta location in the CT stack $I\left( :,:,z_{aa+2} \right)$ (Supplemental Figure 12). The location and the area of the rectangle depends on the location and the size of the ascending aorta in the CT slice. The length of the rectangle was set to 66 pixels based on empirical observations. However, the width ($w, in pixel$) of the rectangle is calculated by

$$w=\left| {\epsilon Ao}_{t}-{\epsilon Ao}_{b} \right|+15$$

where ${\epsilon Ao}_{t}$ is the topmost and ${\epsilon Ao}_{b}$ the bottom most pixel of the ascending aorta region. To ensure that the search space will cover the pulmonary trunk despite inter-individual anatomical variation, we extended the width by 15 pixels. The pixel location (x and y coordinates) of the rectangular is calculated by

$$r\left( y_{min}:y_{max} , x_{min}:x_{max} \right)=\left( {(\epsilon Ao}_{t}-15 \right):{\epsilon Ao}_{b} , \left( {\epsilon Ao}_{l}+5 \right):\left( {\epsilon Ao}_{l}+70 \right) )$$

where ${\epsilon Ao}_{l}$ is the left most pixel of the ascending aorta region. We then draw the rectangle 15 pixels from above from the top point of the ascending aorta and from 5 pixels left of the left most pixel. We calculated 5 and 15 pixels by empirical observation.

After that, we apply the following methods to assign the component with the biggest area as the pulmonary trunk.

- 1. Anisotropic diffusion filtering
  2. Eigen values of hessian matrix
  3. Canny edge detection
  4. Morphological operations

1. **Extracting the pulmonary trunk**

In step 13, we located the pulmonary trunk in the CT stack $I\left( :,:,z_{pt} \right)$. Our main purpose here is to calculate the diameter of the pulmonary trunk in a given CT scan. Calculating the diameter of the pulmonary trunk in several slices gives more accurate and precise results than calculating in one slice. Thus, we tracked the pulmonary trunk starting at the level of the carina trachea anteriorly to the conus arteriosus (infundibulum) in order to reach the pulmonary valve/proximal part of the pulmonary trunk (PT) in the cranial to caudal direction. Anatomically, if we track the anterior areas of the pulmonary trunk in cranial to caudal direction, we can reach the conus arteriosus, where the pulmonary valve/proximal part of the pulmonary trunk (PT) is located. Therefore, we first take a trackable region from the anterior part of the pulmonary trunk (Supplemental Figure 13). We then segment and track the pulmonary trunk slice by slice using the following steps:

- 1. Gray scale segmentation
  2. Anisotropic diffusion filtering
  3. Eigen values of hessian matrix
  4. Canny edge detection
  5. Morphological operations
  6. 2D region growing

1. **Detecting the pulmonary valve/proximal part of the pulmonary trunk (PT)**

In step 14, we segmented and tracked the pulmonary trunk starting at the level of the carina trachea anteriorly to the conus arteriosus (infundibulum) in order to reach the pulmonary valve/proximal part of the PT. To know that the last region we segmented is the pulmonary valve/proximal part of the PT, we check the circularity of the segmented region. To check circularity (or sphericity), we consider the two criteria below.

The first criterion is:

$${cir}_{1}=\frac{4\pi A}{P^{2}}$$

where $A$ and $P$ are the area and the perimeter of the component respectively. The output of the first criterion is between 0 and 1. A component whose ${cir}_{1}$ is 1 is actually a circular object, but since the pulmonary trunk has a complex morphology, ${cir}_{1}$ of the pulmonary valve will not be 1. We empirically determined a ${cir}_{1}$ cut-off value of 0.75 to designate the component as the pulmonary valve/proximal part of pulmonary trunk. If the calculated ${cir}_{1}$ value is < 0.70 we continue to segment and track the pulmonary trunk until we find a circular object. However, if the calculated ${cir}_{1}$ value is between 0.70 and 0.75, the component may not be identified as the pulmonary valve/proximal part of pulmonary trunk. Accordingly, the pulmonary valve/proximal part of pulmonary trunk can be missed by the first criterion.

To find the pulmonary valve/proximal part of pulmonary trunk in case it was missed by the first criterion, we define a second criterion (${cir}_{2}$) consisting of three sub steps. First, we apply the Hough transform to the segmented component to find vertical and horizontal lines inside of the object (Supplemental Figure 14). Second, we search for the longest vertical and horizontal lines by comparing line lengths. Finally, the absolute value difference of the longest vertical and the longest horizontal lines is calculated. If the calculated absolute value difference is < 20 pixels, then the component is designated as a circular object. Theoretically, the length of the major (horizontal) and the minor (vertical) axes of an ellipse is equal. Hence, we have empirically found that the cut-off value 20 is adequate to designate a component as a circular object, and can formulize the circularity checking algorithm below:

$$circularity= \left\{ \begin{aligned} continue tracking, {cir}_{1}<0.7 \\ continue tracking, {( cir}_{1}>0.7 &&&and {cir}_{1}<0.75) and {cir}_{2}> 20 \\ circular, {cir}_{1}\geq0.75 \end{aligned} \right.$$

1. **Extracting the pulmonary valve/proximal part of the pulmonary trunk**

In step 15, we located the pulmonary valve/proximal part of the pulmonary trunk. The last segmented component by Step 15 in the CT slice $I\left( :,:,z_{pv} \right)$ is the pulmonary valve/proximal part of the pulmonary trunk.

**How to Evaluate the Detection Results and What Are the Criteria?**

The marker which was inserted on a CT image by our developed algorithm represents which organ or anatomical landmark we detected. This marker consists of a single pixel also known as seed point. We have resized the marker (becoming filled circle) so that this point can be seen more easily on the image. If the marker hits one of the voxels of the desired organ, then the detection task accomplished. Here, the radiologist's mission is to check if the marker is in the desired organ or not. If it is in the desired organ, then the detection task is labeled as passed otherwise failed.

In this study, we have 7 detection tasks, namely:

Task 1: Detection of the Trachea,

Task 2: Detection of the Bifurcation Point of Airways

Task 3: Detection of the Carina Level,

Task 4: Detection of the Descending Aorta,

Task 5: Detection of the Ascending Aorta,

Task 6: Detection of the Proximal Part of the Pulmonary Trunk/Pulmonary Valve,

Task 7: Detection of the Pulmonary Trunk.

Each task has its own evaluation criteria depending on aim of the detection. You can find in the following sections. But there is a general criterion for failed cases which is described in below:

The blank image means that the algorithm is failed to produce any result (Figure 23).

if you have seen a blank image you can directly labeled as failed!

**Task 1: Detection of the Trachea**

*Evaluation Criteria:* If the marker is inside of the Trachea then, it can be labeled as passed otherwise it must be labeled as failed!

*Example:* The marker is inside the Trachea so in that case, the detection task can be labeled as passed (Figure 24).

**Task 2: Detection of the Bifurcation Point of Airways**

*Evaluation Criteria:* If the two markers are inside of the left and the right main bronchus then, it can be labeled as passed otherwise it must be labeled as failed!

*Example:* The two distinct markers are inside of the left and the right main bronchus so in that case, the detection task can be labeled as passed (Figure 24).

**Task 3: Detection of the Carina Level**

*Evaluation Criteria:* According our observation, if the distance between the left main bronchus and the right main bronchus is bigger than 0.7 cm and smaller than 1.7 cm then we can easily be able to detect the descending aorta. So, in the image you can be able to see the distance, If the distance is between 0.7 cm and 1.7 cm then, it can be labeled as passed otherwise it must be labeled as failed!

*Example:* The distance between the left main bronchus and the right main bronchus is bigger than 0.7 cm and smaller than 1.7 cm so in that case, the detection task can be labeled as passed (Figure 24).

**Task 4: Detection of the Descending Aorta**

*Evaluation Criteria:* If the marker is inside of the Descending Aorta then, it can be labeled as passed otherwise it must be labeled as failed!

*Example:* The marker is inside the Descending Aorta so in that case, the detection task can be labeled as passed (Figure 24).

**Task 5: Detection of the Ascending Aorta**

*Evaluation Criteria:* If the marker is inside of the Ascending Aorta then, it can be labeled as passed otherwise it must be labeled as failed!

*Example:* The marker is inside the Ascending Aorta so in that case, the detection task can be labeled as passed (Figure 24).

**Task 6: Detection of the Proximal Part of the Pulmonary Trunk/Pulmonary Valve**

*Evaluation Criteria:* If the marker is inside a circular/roundish proximal part of the pulmonary trunk/pulmonary valve, it can be labeled as passed otherwise it must be labeled as failed.

*Example:* The marker is inside the circular object which belongs to pulmonary artery system (probably around pulmonary valve) so in that case, the detection task can be labeled as passed (Figure 24).

**Task 7: Detection of the Pulmonary Trunk**

*Evaluation Criteria:* If the marker is inside of the pulmonary artery then, it can be labeled as passed otherwise it must be labeled as failed!

*Example:* The marker is inside of the pulmonary artery so in that case, the detection task can be labeled as passed (Figure 24).

**How to Evaluate the Segmentation Results and What Are the Criteria?**

The main goal of this study is to compare radiologist measurements versus developed algorithm measurements. And we have a numerical data (ground truth) to make a quantitative comparison of radiologist measurements versus developed algorithm measurements and we assessed in the Figure 4 (main figures).

To make a valid measurement, good image segmentation is needed. The good measurement results are enough to estimate that the segmentation results are also good. However, to test the strength of the developed algorithm and to increase the quality of the study, evaluation of the image segmentation is required. The common way to evaluate image segmentation is using quantitative evaluation methods such as Dice Coefficient, Jaccard’s Index, or Pixel accuracy. But all these methods require pixel-wise annotations, and it is time-consuming. Making a pixel-wise annotation of more than one class in a large data set requires overwhelming work. Since we have a large dataset (n=700) and multi-object (n=4) segmentation task, the only remaining option for us is to use the qualitative evaluation method. Here, we defined the evaluation criteria to evaluate image segmentation results.

We presented segmentation results in two ways, using a mask and a boundary box. The mask or boundary box with a tag was inserted on a CT image by our developed algorithm that represents which organ was segmented. The mask contains a set of pixels and the boundary box is a rectangle that covers an object.

For evaluating segmentation results checking the mask or the boundary box is adequate. Here, we encouraged the radiologists to evaluate with the boundary box for fast and easy evaluation. The radiologist's mission is here to check if the boundary box covers the desired organ or not. However, if the radiologists cannot decide that the segmentation is passed or failed by evaluating with the boundary box (for instance the boundary box covers also other organs/tissues), the radiologists can go deeper by evaluating with the mask.

In this study, we have 4 segmentation tasks, namely:

Task 1: Segmentation of the Trachea,

Task 2: Segmentation of the Descending Aorta,

Task 3: Segmentation of the Ascending Aorta,

Task 4: Segmentation of the Pulmonary Trunk.

Each task has its own evaluation criteria depending on aim of the segmentation. The radiologists can find in the following sections.

And there is a general criterion for failed cases which is described in below:

The blank image means that the algorithm is failed to produce any result (Figure 23).

if you have seen a blank image you can directly labeled as failed!

**Task 1: Segmentation of the Trachea**

*Evaluation Criteria:* We have a 3D-Segmentation of the trachea from superior slices of the CT examinations to the bifurcation point. But we only evaluate one of the segmented trachea areas in 3D-segmentation volume.

- If the boundary box covers the majority[[1]](#footnote-2) of trachea then, it can be labeled as passed otherwise it must be labeled as failed!
- If the boundary box over segmented[[2]](#footnote-3) the trachea then, it must be labeled as failed!

*Example:* The boundary box covers the whole trachea, in that case, the segmentation task can be labeled as passed (Figure 25).

**Task 2: Segmentation of the Descending Aorta**

*Evaluation Criteria:*

- If the boundary box covers the majority[[3]](#footnote-4) of descending aorta then, it can be labeled as passed otherwise it must be labeled as failed!
- If the boundary box over segmented[[4]](#footnote-5) the descending aorta then, it must be labeled as failed!

*Example:* The boundary box covers the whole descending aorta, in that case, the segmentation task can be labeled as passed (Figure 25).

**Task 3: Segmentation of the Ascending Aorta**

*Evaluation Criteria:*

We have a 3D-Segmentation of the ascending aorta. But we only evaluate one of the segmented areas in 3D-segmentation volume.

- If the boundary box covers the majority[[5]](#footnote-6) of the ascending aorta then, it can be labeled as passed otherwise it must be labeled as failed!
- If the boundary box over segmented[[6]](#footnote-7) the ascending aorta then, it must be labeled as failed!

*Example 1:* The boundary box covers the whole ascending aorta, in that case, the segmentation task can be labeled as passed (Figure 25).

*Example 2:* The boundary box covers the Estimated 75% or more of the whole area of the ascending aorta and also the boundary box is not clearly over segmented the ascending aorta in that case, the segmentation task can be labeled as passed (Figure 25).

**Task 4: Segmentation of the Pulmonary Trunk**

*Evaluation Criteria:*

We have a 3D-Segmentation of the pulmonary trunk. But we only evaluate one of the segmented areas in 3D-segmentation volume.

- If the boundary box clearly covers the upper (y-axis, in the axial-plane) part of the pulmonary trunk then, it can be labeled as passed otherwise it must be labeled as failed!
- If the radiologists cannot decide that the segmentation is passed or failed by evaluating with the boundary box (for instance the boundary box covers also other organs/tissues), the radiologists can check the mask by following criteria:
  - If the right main pulmonary artery is the only extra structure included in the mask then, it can be labeled as passed!
  - If more distal arterial pulmonary segments, or other vascular or soft tissue structures, are included in the mask then, it must be labeled as failed!

*Example:* The boundary box covers the whole pulmonary trunk, in that case, the segmentation task can be labeled as passed (Figure 25).

1. estimated 50% or more of the whole area [↑](#footnote-ref-2)
2. The box is clearly larger than the trachea [↑](#footnote-ref-3)
3. estimated 75% or more of the whole area [↑](#footnote-ref-4)
4. The box is clearly larger than the descending aorta [↑](#footnote-ref-5)
5. Estimated 75% or more of the whole area [↑](#footnote-ref-6)
6. The box is clearly larger than the ascending aorta [↑](#footnote-ref-7)
